# Integrating new fruit and vegetable growth parameters in SWAT models for improved simulations

**DOI:** 10.3389/fpls.2026.1745017

**Published:** 2026-05-26

**Authors:** Tássia Mattos Brighenti, Natalja Čerkasova, Tiffanie F. Stone, Philip W. Gassman, Jeffrey G. Arnold, James R. Kiniry, Matt Liebman, Manyowa N. Meki, Raghavan Srinivasan, Ajay Nair, Michael J. White, Janette R. Thompson

**Affiliations:** 1 Center for Agricultural and Rural Development, Iowa State University, Ames, IA, United States; 2 Texas A&M AgriLife, Blackland Research & Extension Center, Temple, TX, United States; 3 Klaipėda University, Marine Research Institute, Klaipėda, Lithuania; 4 Department of Agroecology, Aarhus University: Aarhus Universitet institution, Foulum, Denmark; 5 Department of Horticulture, Iowa State University, Ames, IA, United States; 6 Department of Agronomy, Iowa State University, Ames, IA, United States; 7 Grassland Soil and Water Research Laboratory, USDA-ARS, Temple, TX, United States; 8 Department of Natural Resource Ecology and Management, Iowa State University, Ames, IA, United States

**Keywords:** dry yields, fresh yields, harvest index, potential heat unit, SWAT2012, table food

## Abstract

The Soil and Water Assessment Tool (SWAT) includes plant parameters for only a few fruits and vegetables. Testing SWAT parameters for these crops remains very limited, including the need for evaluation of table food crops in historically low production areas. This study tests SWAT simulations for fruits and vegetables in the U.S. Western Corn Belt, to advance model evaluation in a data-limited region. We focus on 24 crops, integrating parameters from related models, expert opinions, and expected versus simulated yields. Parameters for 15 crops in the SWAT database were revised and nine crops were added. The resulting models achieved yield simulation errors within ±37%, a reasonable level of accuracy for underrepresented crops, and statistical tests further supported the robustness of the simulations. Future climate scenario analyses also revealed potential yield impacts and emphasized the need for adaptation strategies. Overall, this study helps fill important gaps in SWAT applications for fruits and vegetables and supports efforts to expand local table food production and crop diversification, although further testing is needed across additional climatic regions and longer crop yield time series.

## Graphical Abstract

**Figure:**
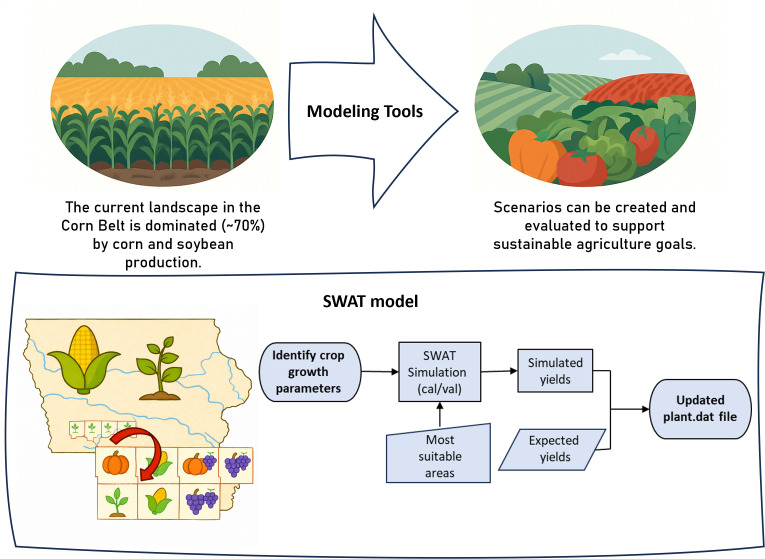

## 1 Introduction

The global prevalence of moderate or severe food insecurity has increased in recent decades, underscoring the fact that maintaining reliable food systems remains a challenge in the 21st century (Allee et al., 2021). This trend highlights the urgency of addressing factors that contribute to food insecurity, such as population growth, climate change, and limited access to resources. In addition, food systems are also a major source of greenhouse gases contributing to climate change impacts (Stone et al., 2021, 2023, 2024; Allee et al., 2021). Based on a global-scale meta-analysis of 570 life cycle assessment studies (median reference year of 2010, beginning with producer inputs and ending at retail) Poore and Nemecek (2018) found food systems at that time created just over 25% of anthropogenic GHG emissions. Diversification of crop production (e.g., diverse rotations, integration of legumes and table food crops, mixed crop-livestock systems) could provide a path toward more sustainable agricultural systems. By understanding the interactions between crops, environmental conditions, and management practices, we can develop more effective strategies to address food production and security (Hernández-Ochoa et al., 2022; Reckling et al., 2023).

The availability of comprehensive data on land area and yields for various crops is crucial for understanding and analyzing agricultural productivity. In the United States, the United States Department of Agriculture (USDA) provides extensive information on land area and yields for many crops. However, fruit and vegetable yield data are limited for the Corn Belt and other regions that produce limited quantities of table food crops, especially in areas that have been historically dominated by agronomic row crop production (Stone et al., 2021, 2023, 2024). This data gap presents a challenge for accurately assessing productivity of these crops on a regional scale, especially given the potential for increasing future demand. One strategy to reduce greenhouse gas emissions in agriculture is by localizing food production (Stone et al., 2023). Additionally, there is an opportunity to expand local table food production to support other nutritional, environmental, and social sustainability goals (Birtalan et al., 2021; Johansson et al., 2014; Edwards-Jones, 2010). This highlights the need for accurate estimates of fruit and vegetable crop yields in regions where data is limited. A key method of generating growth and yield data for various crop types is the use of simulation models (Basso and Liu 2019; Siad et al., 2019; Gaudio et al., 2019). The Iowa UrbanFEWS project (Thompson et al., 2021; Stone et al., 2021, 2023; Brighenti et al., 2022b) applies this modeling framework to identify opportunities for increasing table food production within the six-county Des Moines Metropolitan Statistical Area (DMMSA), the largest metropolitan area in Iowa.

The Soil and Water Assessment Tool (SWAT) is a spatially-oriented, continuous time-step ecohydrological model (Arnold et al., 1998, 2012b; Williams et al., 2008; Neitsch et al., 2011), which can generate water balance, water quality indicators, and crop yield data for current climate conditions and future climate projections. SWAT directly evolved from an interface of the earlier Simulator for Water Resources in Rural Basins (SWRRB) (Williams et al., 1985, 2008; Arnold et al., 1987, Arnold et al., 1990) and the Routing Outputs to the Outlet (ROTO) (Arnold et al., 1995) models; a simplified version of the Environmental Policy Integrated Climate model (EPIC) crop growth sub- model (Williams et al., 1989, 2008; Williams, 1990) was also incorporated. Since the release of SWAT94.2, continuous modifications have refined the ability of the model to simulate crop growth and related impacts. The SWAT model has been used worldwide to evaluate a broad suite of water resource issues for study domains representing an extensive range of environmental conditions and watershed scales (Gassman et al., 2007, 2022; Arnold et al., 2012b; Bressiani et al., 2015; Tan et al., 2019; Brighenti et al., 2019b; Akoko et al., 2021; Gassman et al., 2026).

SWAT incorporates realistic plant growth via parametrization that can assist in developing climate-resilient agricultural practices across various watershed scales. Accurate identification of plant growth parameters allows a better understanding of how crops interact with temperature and water cycles, enabling, for example, the prediction of water requirements and the potential impacts of agricultural activities on water resources (Neitsch et al., 2011; Arnold et al., 2012b). Additionally, the model can simulate various planting and harvesting dates, identify suitable crops for many regions, and describe appropriate irrigation practices, fertilizer applications, agricultural management strategies, and nature-based solutions. SWAT has been used extensively to simulate row (e.g., corn, soybean, sorghum) and close-grown (e.g., wheat, oat, barley, rye) crops, alfalfa and other types of forage crops, pasture and range, and deciduous and evergreen forests (Gassman et al., 2026). However, the model is also increasingly used for simulating other types of vegetation such as planted and native forest (Wang et al., 2022), rubber and oil palm (Heidari et al., 2020; Tan et al., 2021; Bridhikitti et al., 2023), chrysanthemum and rose flowers (Bannwarth et al., 2016), sea buckthorn (Jordan et al., 2018), tea (Lin et al., 2022), coffee (Nkwasa et al., 2020; Msigwa et al., 2022), chat and teff (Nigussie et al., 2019), sugarcane (Meki et al., 2015; Dawit et al., 2020; Bridhikitti et al., 2023), pearl millet (Li et al., 2014; Garg et al., 2021), tobacco (Jamshidi and Naderi, 2023), and additional fruits and vegetables (**Supplementary Material**: **Supplementary Tables 1**, **2**).

The initial establishment of several SWAT plant parameters was documented by Arnold et al. (2012a) for nearly 80 specific crops, grasses and trees, including a subset of fruit and vegetable crops. Further development and testing of plant parameters for various row crops, close-grown crops, grasses and other vegetation has been reported in a variety of studies (e.g., Kiniry et al., 2001; Breuer et al., 2003; Luo et al., 2008; Meki et al., 2015; Yang and Zhang, 2016; Sinnathamby et al., 2017; Alemayehu et al., 2017; Wang et al., 2022). Testing of plant parameters for fruit and vegetable crops in SWAT has been increasing in recent years but is still limited overall and mainly focused on specific production regions. Thus, there is a need for more in-depth testing of fruit and vegetable parameters in SWAT, especially for areas that currently do not feature large-scale production for such crops.

In this study we compiled and tested growth parameters associated with 24 different types of fruit and vegetable crops and integrated them into the SWAT model parameter set. The specific objectives were to: (1) determine expected yields for 24 fruit and vegetable crops, (2) propose SWAT crop parameters for each of the crops tested, (3) compare SWAT-generated simulated yields versus expected crop yields. This is the most extensive set of crop growth and yield results ever reported for fruits and vegetables in any SWAT application to date.

## 2 Materials and methods

### 2.1 Study area

**Figure 1:**
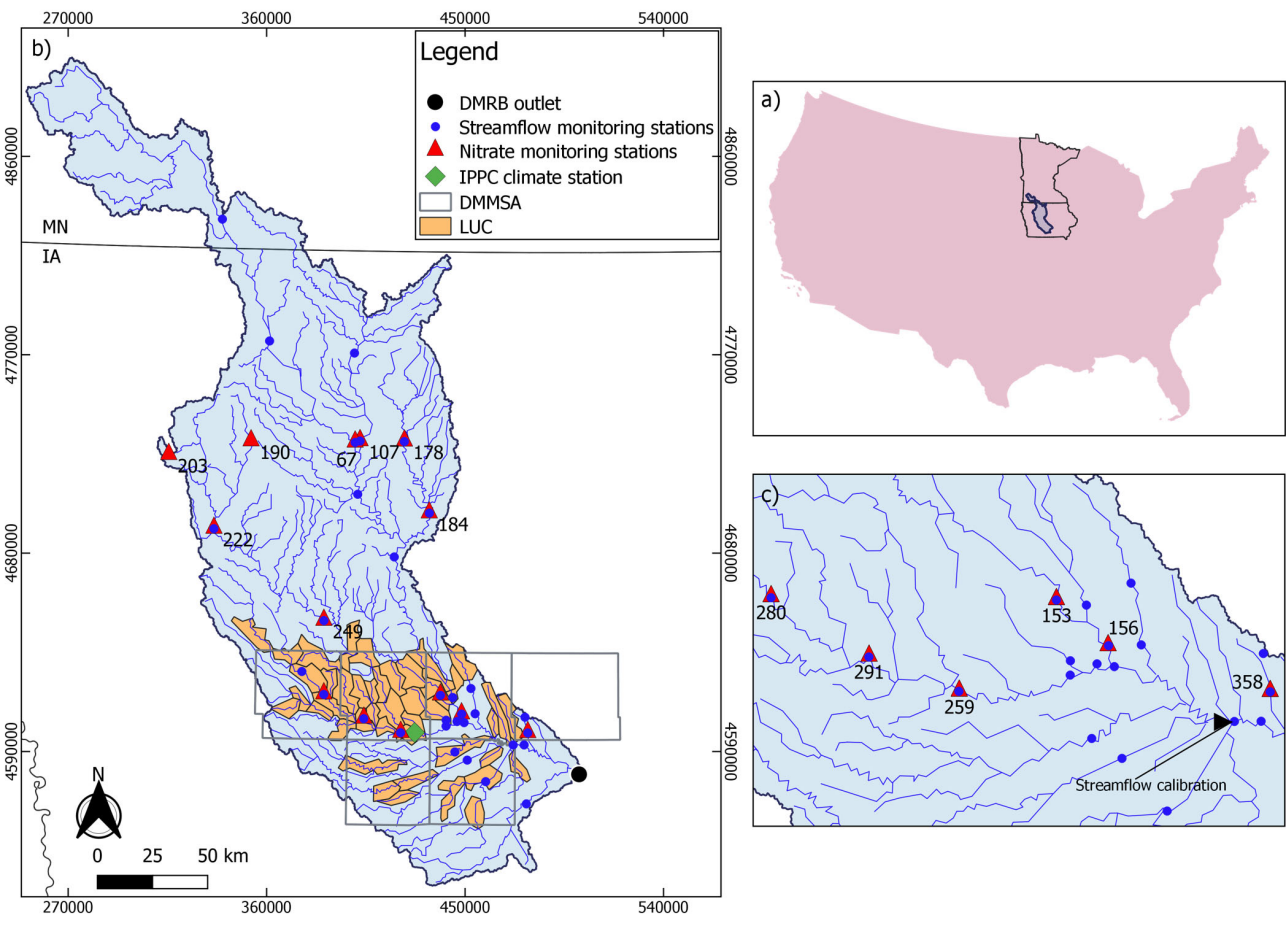
**(A)** Location of the Des Moines River Basin (DMRB) within Iowa and Minnesota, United States, **(B)** streamflow and nitrate monitoring stations, IPPC station for potential heat unit climate data, the Des Moines Metropolitan Statistical Area (DMMSA), and highlighted areas of subbasins where land use changes (LUC) were modeled, **(C)** zoom-in to nitrate and streamflow stations (280, 291, 259, 153, 156, 358) within the DMMSA.

The models described herein were developed as part of a larger project aimed at describing table food production near a metropolitan area (Thompson et al., 2021; Stone et al., 2021, 2023; Brighenti et al., 2022b). Project investigators incorporated multiple models focused on identifying opportunities to increase table food production in the six-county Des Moines Metropolitan Statistical Area (DMMSA), located within the Des Moines River Basin (DMRB). The DMRB study region drains 31,892 km² and extends from southern Minnesota to south-central Iowa, in the Western Corn Belt region of the United States (**Figure 1**). The current land use is dominated by corn and soybean crop systems (~70%). The major soil types are Udolls (freely drained soils within the Mollisol order), Aquolls (wet Mollisols), and Udalfs (Alfisols) (USDA-NRCS, 2022). The basin is predominantly flat, with 13% of the basin having slopes ≥ 4% and ≤ 6%, and the remaining 12% featuring slopes ≥ 6% and ≤ 14%. According to the Köppen classification, the climate is Dfa (Peel et al., 2007), which is characterized by humid continental conditions with hot summers and cold winters. According to USDA census data, an average of 54% of the total landscape is managed with tile drainage (USDA, 2022). The annual average (1985-2018) precipitation is 873 mm, and the reference evapotranspiration (ET) is 670 mm (Brighenti et al., 2022a).

We utilized several datasets obtained via the Hydrologic and Water Quality System (HAWQS) platform (https://hawqs.tamu.edu/) as part of the SWAT simulations. We used a 30-meter resolution Digital Elevation Model (DEM) from the U.S. Geological Survey’s (USGS) (https://www.usgs.gov/the-national-map-data-delivery/gis-data-download), and land use information was sourced from the USGS National Land Cover Database (NLCD) and Cropland Data Layers (CDL) (USGS, 2018; USDA-NASS, 2022). The soil map was derived from the 1:250,000-scale Digital General Soil Map of the United States (STATSGO) (USDA-NRCS, 2022). Weather data (1999 to 2012), including rainfall, temperature, humidity, wind speed, and solar radiation, were obtained from the Parameter-elevation Relationships on Independent Slopes Model (PRISM; PRISM 2022).

Long-term streamflow and nitrate data for the entire watershed were accessed through the USGS database (https://waterdata.usgs.gov/nwis). For streamflow, 25 monitoring gauges were selected, with time series ranging from 2001 to 2010. Additionally, 11 gauges were chosen for nitrate (i.e., computed daily loads), with time series that overlapped from 1999 to 2018 (**Figure 1**) and were accessible through the USGS database, and 3 gauges via Mount et al. (2024) (i.e., daily loads were built from water quality monitoring data and USGS streamflow observations).

### 2.2 Suitable soil classes for fruit and vegetable production

**Figure 2:**
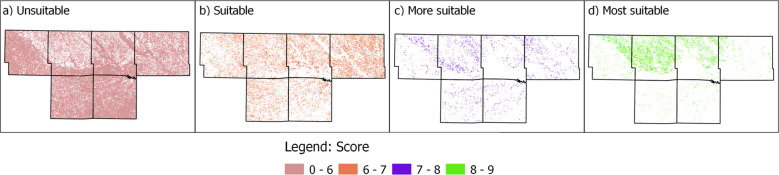
Six-county DMMSA suitable soil classes for fruits and vegetable production based on a scoring system (0 to 9) of the biophysical features: **(A)** unsuitable (0 to 6), **(B)** suitable (6 to 7), **(C)** more suitable (7 to 8), and **(D)** most suitable (8 to 9) (Stone et al., 2024).

The DMMSA served as approximate boundaries for choosing areas to be planted with fruits and vegetables due to: (1) the focus of the larger project (Thompson et al., 2021; Brighenti et al., 2022b; Stone et al., 2024) on the DMMSA (**Figures 1**, **2**) available data indicating areas suitable for growing fruits and vegetables (**Figure 2**). The land area converted to fruit and vegetable production was based on larger project goals (Thompson et al., 2021; Brighenti et al., 2022a), which envision 50% of the table food consumed in the DMMSA being grown locally by the year 2050.

A key aspect of simulating the fruit and vegetable crops in the DMRB was selecting appropriate fields with adequate drainage for crop production. A fruit and vegetable production suitability score for agricultural land (**Figure 2**) was developed based on five criteria with equal weighting based on key biophysical features (Stone et al., 2024). The most suitable land (9 on a scale of 0 to 9) had the following characteristics: (1) zoned agricultural, (2) no flooding, (3) well-drained soil drainage class, (4) slope of 0 – 5%, and (5) soil texture of sandy loam, loam or silt loam. Production of crops across much of Iowa relies on sub-surface tile drainage to remove excess water. Thus, for this parameter, land with a dual drainage class (i.e., well-drained based on tile drainage) was considered equally suitable for selection. Only land parcels with a composite score of 8-9 (most suitable class in **Figure 2**) were considered suitable and selected for fruit and vegetable production in the SWAT model. The resulting appropriate soil types selected for simulating the 24 table food crops were located within a set of subbasins (**Figure 1**).

After overlapping the suitable soil classes map and considering expected yields, land was proposed to be converted (**Table 1**) in the LUC subbasins (**Figure 1**) to fruit and vegetable production in our simulations. The total area required to meet the 50% local production goal of the 24 fruit and vegetable crops was only 95.6 km^2^ (9,560 ha), underscoring that these table food needs could be met with a relatively small crop production area, largely due to the highly productive soils in the region and climate conditions particularly amenable for crop growth.

**Table 1:** Iowa expected yields (fresh weight) for the 24 crop types selected, land area to reach 50% of dietary needs, and data source; water content for the crop types selected; and conversion of observed fresh yields into observed dry yields.

Crop type	Iowa yields (ton/ha); land area (km²); Source	Water content (%)	Dry yield (ton/ha)
Apple	30.5; 9.1; ISU Horticulture^1^	83^4^	5.2
Blueberry	6.1; 4.3; ISU Horticulture^2^	80^5^	1.2
Broccoli	17.4; 2.6; ISU Horticulture^3^	91^6^	1.6
Cabbage	62.7; 1.0; ISU Horticulture^3^	93^6^	4.4
Carrot	62.7; 1.2; ISU Horticulture^3^	88^6^	7.5
Cherry	8.8; 0.7; ISU Horticulture^1^	20^7^	7.1
Collard Greens	14.8; 0.4; ISU Horticulture^3^	94^6^	0.9
Corn, Sweet	19.0; 2.1; ISU Horticulture^3^	76^6^	4.6
Cucumber	20.1; 2.2; ISU Horticulture^3^	96^6^	0.8
Dry bean	2.0; 27.8; ISU Horticulture^3^	15^6^	1.7
Grape	17.7; 9.6; ISU Horticulture^2^	78^8^	3.9
Kale	30.9; 0.1; ISU Horticulture^3^	85^6^	4.6
Lettuce	40.2; 2.2; ISU Horticulture^3^	94^6^	2.2
Melon	29.9; 6.6; ISU Horticulture^3^	90^6^	3.0
Onion	59.7; 2.4; ISU Horticulture^3^	91^6^	5.4
Pear	35.2; 1.4; ISU Horticulture^1^	83^6^	6.0
Potato	45.2; 7.4; ISU Horticulture^3^	79^6^	9.5
Pumpkin	35.9; 0.4; ISU Horticulture^3^	92^6^	2.9
Raspberry	7.2; 2.1; ISU Horticulture^2^	85^4^	1.1
Spinach	18.8; 0.9; ISU Horticulture^3^	92^6^	1.5
Squash	21.9; 1.5; ISU Horticulture^3^	91^6^	2.1
Strawberry	58.7; 3.7; ISU Horticulture^2^	92^6^	4.7
Sweet Potato	24.8; 1.4; ISU Horticulture^3^	76^6^	6.0
Tomato	90.3; 4.5; ISU Horticulture^3^	94^6^	5.4

^1^ Personal Communication 2022, Slack & Nonnecke; ^2^ Personal Communication 2022, Nonnecke; ^3^ Personal Communication 2022, Nair; ^4^ source Hackett and Carolane, 1982, ^5^ source Gonçalves et al. (2015); ^6^ source Donald and George (2007); ^7^ source Kandala et al. (2013); ^8^ source Doymaz (2012).

### 2.3 Methodological approach

The fruit and vegetable crops selected for this study can be grown in Iowa and are among the most commonly consumed according to the USDA “food availability per capita” dataset (USDA-ERS, 2023). More information about Iowa food production has been published elsewhere (Thompson et al., 2021; Stone et al., 2024). In general, these crops can also be grown throughout the Corn Belt region. We compiled and evaluated growth parameters for 24 specific crops: apple, blueberry, broccoli, cabbage, carrot, cherry, collard greens, cucumber, dry bean, grape, kale, lettuce, melon, onion, pear, potato, pumpkin, raspberry, spinach, squash, strawberry, sweet corn, sweet potato, and tomato.

**Figure 3:**
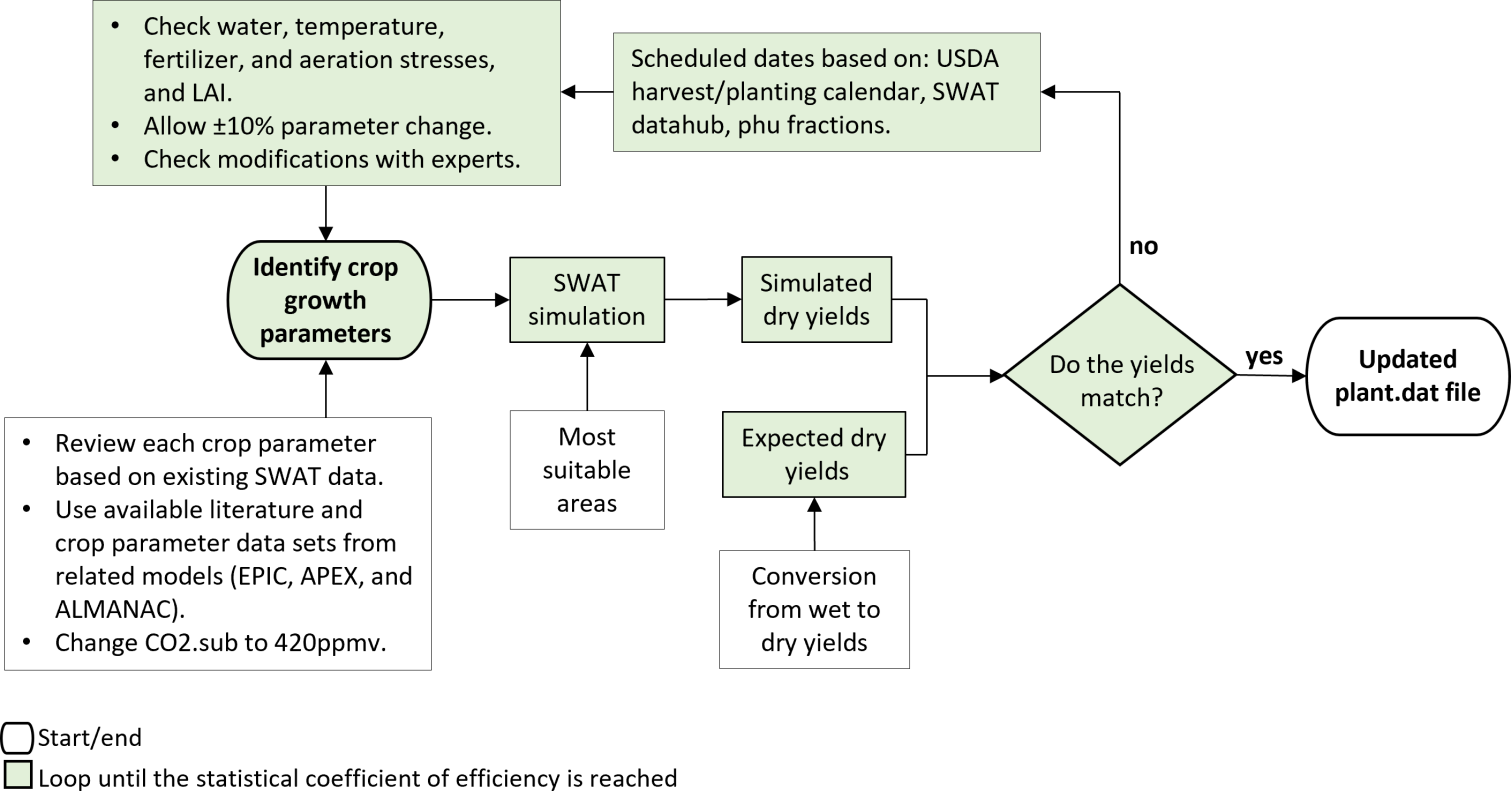
Methodological approach for the fruit and vegetable crop growth parameter evaluation.

We followed a stepwise methodological approach (**Figure 3**). The first step in identifying the crop growth parameters for a given crop involved reviewing the existing SWAT crop parameter database, and then reviewing published literature and databases from three models closely related to SWAT: the Erosion-Productivity Impact Calculator (EPIC), the Agricultural Policy/Environmental eXtender (APEX) model (Williams et al., 2008; Gassman et al., 2010; Steglich et al., 2019), and the Agricultural Land Management Alternative with Numerical Assessment Criteria (ALMANAC) model (Kiniry et al., 1992; Williams et al., 2008). These two steps (i.e., searching literature and examining existing crop parameters) were responsible for initially filling in the necessary parameters for each of the crops. It is important to note that previous testing of many of the available fruit and vegetable crop parameters is extremely limited.

During the compilation of the parameters, it was discovered that several crops were parameterized in multiple models (e.g., apple, cabbage, carrots are in the databases of SWAT, APEX and EPIC). Variations in at least some of the parameter values typically existed between the models for a given crop parameter set (e.g., apple). In these cases, the model was run for each model parameter set for the specific crop, and the best parameter set was selected based on expert communication. In addition, the CO2.sub parameter, which represents the concentration of carbon dioxide in the atmosphere, was updated from 330 ppmv (current SWAT default value) to 420 ppmv to match current atmospheric conditions (NOAA, 2021).

We built in a threshold error level (Section 2.5.3), based on percent bias (Pbias) statistic, to indicate if the simulated yields matched the expected yields. In the case of exceeding the threshold, parameters used in the crop growth simulation were re-evaluated (and changed if necessary) based on: (1) updates to the scheduled dates of planting and harvest, (2) output file analyses for water, temperature, fertilizer, and aeration stresses, (3) leaf area index (LAI) and potential heat units’ fractions (phu) present during the simulation time, (4) ± 10% allowable epistemic error for change for a given plant parameter relative to the original value, and (5) further checks of each modification with experts (**Figure 3**).The Pbias was calculated as the percentage difference between the simulated crop yield (*yields_sim_*) and the corresponding expected dry yield (*yields_exp_*) over the simulation period:


$$Pbias=100*\frac{\sum \left(yields_{sim,i}-yields_{exp,i}\right)}{\sum yields_{exp,i}}$$

Tests of the 24 fruit and vegetable crop parameters were limited to manual calibration due to a lack of long-term measured yield data. However, the baseline SWAT model was evaluated for long-term streamflow and nutrient data. The SWAT model was executed from 2000 to 2012; therefore, simulated crop yields presented are an average of the 13-year study period. The simulated yields are compared to corresponding “expected dry yields” (see sections 2.3), and the differences are calculated accounting for Pbias values (Section 2.5.3). Expected yields are emphasized in this analysis due to a lack of actual observed fruit and vegetable yields in the study region, as described in more detail below.

### 2.4 Expected yields

Average yield values for fruit and vegetable production in the state of Iowa are not collected by the United States Agricultural Census because they make up a very small proportion of total agricultural production in the DMMSA (0.2% of cropland; Stone et al., 2024) and other parts of Iowa. In addition, average yields based on national or regional data do not accurately represent Iowa fruit and vegetable production due to differentiating factors (i.e., production scale, mechanization, and soil and climatic conditions; Enderton et al., 2017). Consequently, adjustments were made to existing data to match scale and production for Iowa fruit and vegetable expected yields.

National and Midwest average values were collected as starting points, and expert input was then used to better reflect Iowa-specific production practices and conditions. In general, for Iowa fruit expected yields, national average yields (Peters et al., 2016) were adjusted for Iowa production based on state FSA estimates in combination with input from two fruit crop specialists (Nonnecke and Slack, 2022) as described in Stone et al. (2023b). The expected Iowa vegetable yields were adjusted from a national-scale CleanMetrics FoodPrint LCA Model (CleanMetrics, 2020) that compiled average yields (2000 to 2010) from the United States Agricultural Census (Peters et al., 2016) on a percentage basis to account for differences in Iowa vegetable production using available Midwest yield values (Egel et al., 2020) in combination with expert opinion (Stone et al., 2021).

For example, the national average for apples is 29108.5 kg/ha versus an estimated Iowa average of 27595.4 kg/ha; the Iowa data was based on a 3-year state USDA Farm Service Agency (FSA) average, similar to findings reported by Gleason et al. (1994) and validated via personal communication. For pears, the national average yield is 32560.73 kg/ha, which is close to Iowa’s 30935.49 kg/ha yield. This Iowa estimate was made based on farmer’s reported yields and an Iowa research station typically harvesting 40.8 kg per tree at 3.7-meters spacing between trees (Nonnecke and Slack, 2022). For cherries, both national and Iowa estimated yields are approximately 8002.9 kg/ha, with Iowa’s yield assumed to match the national average due to the local advantage of growing only tart varieties, while the national average includes both tart and lower-yielding sweet varieties. For grapes, the national average yield is 16812.8 kg/ha versus an estimated Iowa yield of 4483.4 kg/ha. The typical Iowa grape yields fall between 2241.7 and 4483.4 kg/ha, while excellent yields are in the 6725.1 to 8966.8 kg/ha range. The FSA 3-year average for grapes is 9258.2 kg/ha (Nonnecke and Slack, 2022). We thus chose the 4483.4 lg/ha yield due to our consideration of table grapes only and expert input.

All yield estimates are for marketable yields based on average varieties/cultivars using “typical” production practices in Iowa in an average climatic year. Due to requirements for an unblemished appearance, marketable yields were lower for fresh fruits and vegetables than for preserved or processed fruits and vegetables (e.g., jelly, salsa) (Stone et al., 2023). Thus, we developed a set of expected yields (fresh weight) used in this study (**Table 1**).

The water content of fruits and vegetables can vary significantly depending upon the crop variety. The initial information available for the Iowa expected yields was reported as fresh (wet) yields (**Table 1**), which were converted to a dry basis to be consistent with crop yields reported by SWAT.

Long-term measured yields and yield distributions or ranges were not available for the 24 fruit and vegetable crops studied. Consequently, it was not possible to analyze measured yields or perform auto-calibration. This contrasts with the streamflow auto-calibration, as reported by Brighenti et al. (2023a) and nitrate calibration conducted in this study, which utilized dozens of monitoring data points. Instead, simulated crop yields were compared to expected yields, representing the most realistic estimates for these crops under central Iowa conditions. This approach is an initial framework for evaluating table food crops in Iowa and other regions similar to the Corn Belt. It highlights the importance of these crops in future cropping systems and emphasizes the critical need for long-term yield data collection to enable more detailed analyses of fruit and vegetable production.

### 2.5 SWAT model configuration and verification

The SWAT model land phase of the hydrological cycle is based on a water balance equation that is calculated for each Hydrological Response Unit (HRU). HRUs serve as the basic model simulation unit in SWAT, which are defined as homogeneous areas comprised of unique soil, land use, slope, and management within a given subbasin (Arnold et al., 1998; Neitsch et al., 2011). The water balance equation is:


$$SW_{t}=SW_{0}+\sum_{i=1}^{t}\left(R_{day}-Q_{surf}-E_{a}-W_{seep}-Q_{gw}\right)$$

where, *SW_t_* is the final soil water content (mm H2O), *SW*_0_ is the initial soil water content on day i (mm H2O), t is the time (days), *R_day_* is the amount of precipitation on day i (mm H2O), *Q_surf_* is the amount of surface runoff on day i (mm H2O), *E_a_* is the amount of ET on day i (mm H2O), *Q_seep_* is the amount of water entering the vadose zone from the soil profile on day i (mm H2O), and *Q_gw_* is the amount of return flow on day i (mm H2O).).

The SWAT plant growth formula is based on daily accumulation of heat units and is computed by subtracting the daily base temperature (T_BASE) from the average temperature (T_AV), when T_AV > T_BASE. Crop growth will only occur on those days where the mean daily temperature exceeds the T_BASE. Plant development is dependent on temperature, water, nitrogen and/or phosphorus stress, operation management timing, leaf area development, light interception, and conversion of intercepted light into biomass assuming a plant species-specific radiation-use efficiency. The model categorizes plants into seven different types: warm season annual legume, cold season annual legume, perennial legume, warm season annual, cold season annual, perennial and trees (Neitsch et al., 2011). Biomass accumulates during the growing season (planting to harvest/dormancy) on days when the mean temperature exceeds the plant base temperature and continues until the crop maximum heat units are reached (Khalili et al., 2021). The actual biomass is simulated by accounting for water and temperature stress, and nutrient limitation during the plant growth simulation. When the harvesting process is executed by the harvest index (HVSTI), a portion of the plant biomass is removed as yield. For aboveground crops the harvest index will be less than one, for belowground crops (e.g., onions, potatoes) the harvest index may be greater than one. The remaining fraction of the crop, after harvest, is converted into residue (Neitsch et al., 2011). The crop yield in the model is calculated as:


$$yld=bio_{ag}*\text{HVSTI when HVSTI}\leq 1.00$$

$$yld=bio\left(1-\frac{1}{\left(1+\text{HVSTI}\right)}\right)\text{ when HVSTI}>1.00$$

$$bio_{ag}=(1-fr_{root})bio$$

where yld is the crop yield (kg/ha), *bio_ag_* is the aboveground biomass on the day of harvest (kg/ha), HVSTI is the harvest index on the day of harvest, and bio is the total plant biomass on the day of harvest (kg/ha) and *fr_root_* is the fraction of total biomass in the roots on the day of harvest.

The SWAT2012 model (Version 2012 Revision 685) was configured using the Penman-Monteith equation to calculate potential ET and the variable storage coefficient method to calculate the channel flood routing routine. An alternative runoff curve number (RCN) approach was used to calculate the surface runoff [Kannan et al., 2008; Arnold et al., 2012b; Brighenti et al. (2022a, 2023a)]. This RCN method calculates the retention parameter as a function of ET using a CN coefficient (CNCOEF = 0.75). The operation management schedule was established based on dates, and the SWAT auto-fertilization routine was used to simulate nitrogen fertilizer inputs; irrigation was not implemented. The simulation time period, 1998 to 2012 with a 2-year warm-up period, was established in accordance with the time frame used by the CleanMetrics FoodPrint Model (2000 to 2010) and the land use map from the HAWQS platform setup (2006 to 2012) (Peters et al., 2016; HAWQS, 2020).

#### 2.5.1 Automatic calibration for streamflow and nitrate

The baseline SWAT model configuration and hydrological testing used in this study are detailed by Brighenti et al. (2022a, 2023a). In this section, we highlight key aspects of the calibration methodology proposed by Brighenti et al. (2023a), along with additional steps taken in model evaluation, such as a calibration analysis of nitrate data.

The baseline SWAT model was calibrated applying the SUFI-2 optimization algorithm (Abbaspour et al., 2004) to simulate streamflow in the DMRB from 2001 to 2010. The calibration focused on adjusting several hydrological parameters, such as runoff curve number, distance between tile drains, groundwater delay, and soil lateral saturated hydraulic conductivity factor to improve the model’s accuracy in replicating observed streamflow. The calibration was done at both monthly and daily time scales using observed streamflow data from a primary gauge near the basin outlet. The results were then validated across 24 upstream monitoring stations to test the model’s spatial reliability (Brighenti et al., 2023a). These simulations were performed with typical row crop systems consisting primarily of corn and soybean (see Brighenti et al., 2022a for a comparison of simulated DMRB corn biomass and grain yields versus USDA-National Agricultural Statistics Services (NASS) survey data).

For nitrate evaluation, the baseline for the model (Brighenti et al., 2022a) was updated to include manure and commercial fertilizer applications. The manure was spatially distributed across subbasins in the DMRB and applied to corn fields. Nitrogen and phosphorus application rates (kg/ha) were calculated for each subbasin based on the animal feeding operations located within it.

Data on animal feeding operations were collected from geospatial databases for Iowa (https://geodata.iowa.gov/) and Minnesota (https://gisdata.mn.gov/), encompassing medium and large-scale facilities. Each facility’s animal units were converted into actual animal numbers using specific equivalency factors (i.e., animal units = 1; swine = 0.4; cattle = 1.0; layer chicken = 0.01). To calculate the quantities of nitrogen and phosphorus in manure, the total number of animals was multiplied by the manure production rates for each livestock type, with adjustments made for typical nitrogen losses (Libra et al., 2004). The manure’s inorganic and organic nutrient fractions were calculated, accounting for nitrogen transformation processes like ammonium formation (Gassman et al., 2002).

In addition, an initial automatic calibration was performed. Nitrate data from the 14 monitoring stations were analyzed in SUFI-2 (Sequential Uncertainty Fitting ver. 2) using the optimal parameter set from streamflow calibration. The SWAT model was run for 400 iterations, and the best-performing nitrate simulation was documented with statistical evaluation (**Table 2**).

**Table 2:** Parameters that varied during NO3 loads calibration, ranges and final values.

Parameter	Range		Final
	Min.	Max.	
CH_K2.rte (effective hydraulic conductivity in main channel alluvium)	0	500	343
ERORGN.hru (organic N enrichment ratio)	0	5	4.6
NPERCO.bsn (nitrogen percolation coefficient)	0.01	1	0.28
RCN.bsn (concentration of nitrogen in rainfall)	0	2.5	2.2
CMN.bsn (rate factor for humus mineralization of active organic nitrogen)	0.0003	0.003	0.001
SOL_NO3(1).chm (Initial NO3 concentration in the first soil layer)	0.5	3	1.7
SOL_AWC(1).sol (Available water capacity of the soil layer)	-0.25	0.25	0.21

Monthly performance for the model was evaluated using the following statistical metrics: Nash-Sutcliffe Efficiency (NSE) (Nash and Sutcliffe, 1970), Percent Bias (Pbias) (Gupta et al., 1999), Coefficient of Determination (R²) (Moriasi et al., 2015), and Kling-Gupta Efficiency (KGE) (Gupta et al., 2009). The NSE was used as the objective function during the calibration process. The NSE evaluates how well the simulated data match the observed streamflow data, with values ranging from 1 (perfect fit) to negative values indicating poor performance. A value ≥ 0.5 is considered satisfactory for hydrological models (Moriasi et al., 2015). Pbias was used to assess the model’s tendency to overestimate or underestimate streamflow. Pbias values close to zero indicate a balanced model, with positive values showing underestimation and negative values showing overestimation (Gupta et al., 1999; Moriasi et al., 2015). The R² measures the linear relationship between observed and simulated data; a value closer to 1 indicates a strong correlation (Moriasi et al., 2015). The formulation for the objective functions is described by Brighenti et al. (2022a, 2023a).

#### 2.5.2 Crop parameter definition

The process of identifying the most accurate value for each plant parameter was initiated by comparing the existing SWAT plant parameters with corresponding parameters available in the EPIC, APEX and ALMANAC plant parameter databases. Differences in values for the same parameters between the SWAT data set and one or more of the other models resulted in subsequent model testing for the specific parameter values as part of the overall testing of the complete plant parameter data sets. Alternative values reported in existing literature or identified via modeling team expertise were also incorporated into the model testing process. Multiple sources of data for the plant parameters were used (**Table 3**).

**Table 3:** Sources of data for sets of plant parameters for each of the 24 fruit and vegetable crops.

Crop (SWAT code)	IDC^1^	Primary model data set source	Modified plant parameters based on model testing and other data sources^2^
Apple (APPL)	7	SWAT	BIO_E (Hackett and Carolane, 1982; and ±10% change); HVSTI (± 10% change); T_OPT (APEX & EPIC); T_BASE (Hackett and Carolane, 1982; Donald and George, 2007).
Blueberry (BLUE)	6	APEX	WSYF, WAVP, HVSTI (modeling team expertise); T_OPT & T_BASE (Hackett and Carolane, 1982).
Broccoli (BROC)	5	SWAT	BIO_E, HVSTI, BLAI, FRGRW1, FRGRW2, LAIMX1, LAIMX2, DLAI, T_OPT, T_BASE and WSYF (Kim et al., 2021; and modeling team expertise).
Cabbage (CABG)	5	SWAT	BIO_E, FRGRW1, FRGRW2, LAIMX1, LAIMX2, DLAI, CHTMX, T_OPT, T_BASE and WSYF (Kim et al., 2020; and modeling team expertise).
Carrot (CRRT)	4	SWAT	BIO_E, BLAI, WSYF (± 10% change); HVSTI (El-Helaly, 2018); T_BASE (Hackett and Carolane, 1982).
Cherry (CHER)	7	SWAT^3^	T_OPT & T_BASE (Hackett and Carolane, 1982).
Collard greens (COLG)	5	APEX	No further modifications.
Cucumber (CUCM)	4	SWAT	CNYLD, PLTNFR (1), PLTNFR (2), PLTNFR (3), GSI & ALAI_MIN (EPIC); BIO_E (± 10% change); HVSTI, WSYF (modeling team expertise).
Dry bean (DRYB)	1	EPIC	HVSTI, FRGRW1, FRGRW2, LAIMX1, DLAI, T_OPT & T_BASE (Kim et al., 2020).
Grape (GRAP)	6	SWAT	HVSTI & WSYF (modeling team expertise).
Lettuce^4^ (LETT)	5	SWAT	HVSTI, RDMX, T_OPT, T_BASE, CNYLD, PLTNFR (1), WSYF, GSI & ALAI_MIN (EPIC); BIO_E & BLAI (± 10% change).
Kale (KALE)	5	APEX	No further modifications.
Melon^4^ (HMEL)	4	SWAT	BIO_E (± 10% change); HVSTI (Colla et al., 2010); CHMTX, RDMX, CNYLD, PLTNFR (1), PLTNFR (2), PLTNFR (3), GSI, WAVP, CO2HI & ALAI_MIN (EPIC); T_BASE (Hackett and Carolane, 1982); WSYF (modeling team expertise).
Onion (ONIO)	5	SWAT	CHMTX, RDMX, PLTNFR (1), PLTNFR (2), PLTNFR (3), GSI & ALAI_MIN, T_OPT (EPIC); T_BASE (Hackett and Carolane, 1982; Donald and George, 2007).
Pear (PEAR)	7	SWAT^3^	BIO_E, HVSTI (± 10% change); T_OPT and T_BASE (Hackett and Carolane, 1982).
Potato (POTA)	4	SWAT	BIO_E, BLAI, CHMTX, RDMX, T_OPT, CNYLD, CPYLD, PLTNFR (1), GSI, WAVP & ALAI_MIN (APEX & EPIC); T_BASE (Hackett and Carolane, 1982); HVSTI & WSYF^5^
Pumpkin (PUMP)	4	APEX	No further modifications.
Raspberry (RASP)	6	SWAT^3^	HVSTI (modeling team expertise).
Spinach (SPIN)	5	SWAT	CHMTX, RDMX, CNYLD, PLTNFR (1), GSI, WAVP and ALAI_MIN (EPIC); T_BASE (Hackett and Carolane, 1982).
Squash (SQUA)	4	APEX^6^	BIO_E, HVSTI, FRGRW1, FRGRW2, LAIMX1, LAIMX2, DLAI, CHTMX & T_OPT (Kim et al., 2020); T_BASE (Hackett and Carolane, 1982; Donald and George, 2007).
Strawberry (STRW)	4	SWAT	BIO_E & HVSTI (± 10% change); T_BASE (Hackett and Carolane, 1982; Donald and George, 2007); CHTMX, RDMX, GSI & ALAI_MIN (EPIC).
Sweet corn (SCRN)	4	SWAT	HVSTI, BLAI, LAIMX1, T_OPT, WSYF, WAVP & ALAI_MIN (EPIC); T_BASE (Hackett and Carolane, 1982; Donald and George, 2007).
Sweet potato (SPOT)	4	SWAT	BLAI, LAIMX1, T_OPT, PLTNFR (1), PLTNFR (2), PLTNFR (3), PLTPFR (1), PLTPFR (2), WSYF, GSI & ALAI_MIN (EPIC); HVSTI & WSYF^5^
Tomato (TOMA)	4	SWAT	CHMTX, RDMX, T_OPT, CPYLD, GSI, WAVP, ALAI_MIN & BIO_LEAF (EPIC); HVSTI (Cavero et al., 1999).

†^1^ Definitions of IDC (Land cover/plant classification) categories [41]: 1 = warm season annual legume; 2 = cold season annual legume; 3 = perennial legume; 4 = warm season annual; 5 = cold season annual; 6 = perennial; 7 = trees; ^2^ Variable names described in **Table 6** and **Appendix A; Table A1**; ^3^ Based respectively on the SWAT plant parameters for orchard crop (cherry), apple (pear) and grape (raspberry), plus any additional modifications noted in the table; ^4^ Lettuce and melon parameters represent Iceberg and Honeydew varieties, respectively; ^5^ The HSVTI and WSYF values were both changed from 0.95 to 1.05 (potato) and from 0.60 to 1.10 (sweet potato) to more accurately account for above ground biomass effects; ^6^ Based on APEX plant parameters for pumpkin. The complete parameter descriptions can be found in **Supplementary Material**, **Supplementary Table 3**.

The Land cover/plant classification (IDC) served as the primary source of plant parameters. The IDC provides identification of each crop within broader vegetation categories (**Table 3**, footnote 1). The original SWAT plant parameters provided the basis for most of the final sets of parameters (**Table 3**) including substituting orchard crop parameters for cherry, apple parameters for pear and grape parameters for raspberry (due to lack of existing parameters among the models and literature for those three crops). APEX plant parameters were used for four crops (collard green, kale, pumpkin and squash) and EPIC plant parameters served as the source of dry bean parameters (**Table 3**). ALMANAC plant parameters were ultimately not used to determine any of the 24 table food crop parameters for the final SWAT data set.

EPIC parameters were used to adjust one or more final parameter values for eleven crops that were initially configured with SWAT parameters (**Table 3**): apple, cucumber, lettuce, melon, onion, potato, spinach, strawberry, sweet corn, sweet potato and tomato. APEX parameters were also used to adjust selected apple and potato parameters. Literature review and modeling team expertise were further relied on to modify/check most of the plant parameters, and the ±10% change criterion was used to finalize parameter subset values for five crops (apple, carrot, cucumber, lettuce and strawberry). No further modifications were made to the three APEX plant parameter data sets representing collard green, kale and pumpkin.

Growing season date adjustments were performed simultaneously with the other modifications. Due to a lack of specific planting and harvesting time periods for these crops in Iowa, the planting/harvest schedule dates were estimated based on: (1) the USDA harvest/planting calendar (USDA-NASS, 2007) from nearby states (e.g., Minnesota, Illinois, and Michigan), (2) the soil and water datahub (White et al., 2016), (3) potential heat unit fractions reported in the operation management output file (output.mgt), and/or (4) the growing season range (Hackett and Carolane, 1982). Depending on under- or over-estimation of yields, the initial schedule was adjusted, considering the time limits for plant maturation and the temporal scale consistent with the Iowa growing calendar. **Table 3** summarizes, for each crop, the primary source of the original plant parameter set (SWAT, EPIC, or APEX) together with the parameters that were modified during the testing process and the associated literature or expert sources used to justify those adjustments.

The logic behind the heat unit (HU) theory is that plants have heat requirements that can be quantified and linked to the plant’s time to maturity (Neitsch et al., 2011). We calculated heat units (**Table 4**) using a degree-day model where the inputs are the optimal (T_OPT) and minimum (T_BASE) temperatures of each crop type, growing season range, and a temperature climate time series. The IPPC-Degree Day Models Tool (OSU, 2022) allows selection of a climate station for a specific study location and calculates the HU for a given year. We used a 10-year HU average for a climate station located at the center of the DMMSA (**Table 4**, **Figure 1**). The T_OPT and T_BASE values, and growing season range, were the same as those used during the 12-year (2000 to 2012) SWAT simulation.

**Table 4:** Ten-year averages for potential heat units considering a climate station within the DMMSA.

Crop variety	Heat unit (HU) 10-year average	Crop variety	Heat unit (HU) 10-year average
Apple	1129	Lettuce	1603
Dry Beans	1756	Melon	2186
Blueberries	948	Onions	3827
Broccoli	551	Pears	1256
Cabbage	790	Potatoes	925
Carrots	2288	Pumpkin	898
Cherries	1533	Raspberries	1893
Collard greens	1453	Spinach	539
Sweet corn	2341	Squash	719
Cucumber	1505	Strawberries	1268
Grapes	1205	Sweet potatoes	989
Kale	1290	Tomatoes	1689

#### 2.5.3 Statistical evaluation for crop yields

Information on the simulation errors is important in models used to support management decisions (Efstratiadis and Koutsoyiannis, 2010) and the total error allowed by a simulation can be determined by propagating the known potential error sources (Refsgaard et al., 2007). The potential error sources identified for this study were expected yields, water content, and modeling errors.

When evaluating SWAT models, criteria for satisfactory simulations for outputs such as streamflow, suspended sediments, and nutrients are well established (Moriasi et al., 2007, 2015). However, when evaluating fruit and vegetable crop yields, identification of target values is not straightforward, mainly due to the absence of long-term data series. In Iowa, long-term data series for crop yields are available only for selected crops (e.g., corn and soybean) via United States Agricultural Census data (USDA-NASS, 2023). Previous studies (Stöckle and Kemanian, 2020; Čerkasova et al., 2023) reported that yield simulation relative error for these crops can range from ±5% to ±30%. The higher errors are attributed to variable rainfall, irrigation needs, and temperature fluctuations, which complicate crop yield predictions. Data limitations and crop misclassifications can also contribute to error estimations (Čerkasova et al., 2023).

We define an error of ±25% for the expected fruit and vegetable yields based on modeling expertise and experts’ knowledge. The second source of error we identified were differences in the literature values for water content (**Table 1**). Based on that, we specified an error of ±5% to be associated with this process (i.e., conversion of fresh to dry yields). The error associated with the model simulation is based on previous analysis of the DMRB water balance calculation, which was found to be ±27% based on statistical comparisons (using Pbias) for streamflow (Brighenti et al., 2022a).

The Pbias (Gupta et al., 1999) was the statistic used to measure the difference between measured and simulated crop yields. The acceptable range (Ep) is a composition of Pbias values for comparing the observed and simulated yields and is defined by the equation developed by Topping (Topping, 1972; Moriasi et al., 2015). This formulation is usually accepted for grouping and propagating error (Harmel et al., 2006; Bonumá et al., 2013). The equation output is a modified error term that adds independent potential error sources (expected yields E_1= ± 25%, water content E_2= ± 5%, and modeling errors E_3= ± 27%) by incorporating the distribution of measurement uncertainty (Harmel et al., 2006; Rodrigues et al., 2015). Thus, the total probable error according to Equation 6, determined as a function of these three errors, was found to be Ep = ± 37%. This was the maximum permissible error considered when evaluating the simulated yields relative to the expected yields as calculated using:


$$Ep=\sqrt{\sum_{i=1}^{n}\left(E_{1}^{2}+E_{2}^{2}+E_{3}^{2}+\ldots +E_{n}^{2}\right)}$$

where *Ep* is the probable error range; *n* is the number of potential error sources; and *E*_1_, *E*_2_, *E*_3_ are the errors associated with each potential source: expected yields, water content, and modeling errors.

## 3 Results

### 3.1 Streamflow and nitrate analysis

To test the parameters of the 24 crop types, we utilized the previously described SWAT model for the DMRB (Brighenti et al., 2022a, 2023a), which has been continuously updated, and the set of expected yields (**Table 1**). The streamflow calibration performed by Brighenti et al. (2023a) demonstrated that the physically-based tile-drain method provided improved simulation of hydrological processes compared to the empirical method. The physically-based tile drain method also demonstrated more consistent results across the multiple monitoring stations, reflecting improved spatial representation of streamflow. The flow duration curves, and uncertainty analyses confirmed that the modified method provided a more realistic simulation of peak flow and overall water balance in the DMRB.

**Figure 4:**
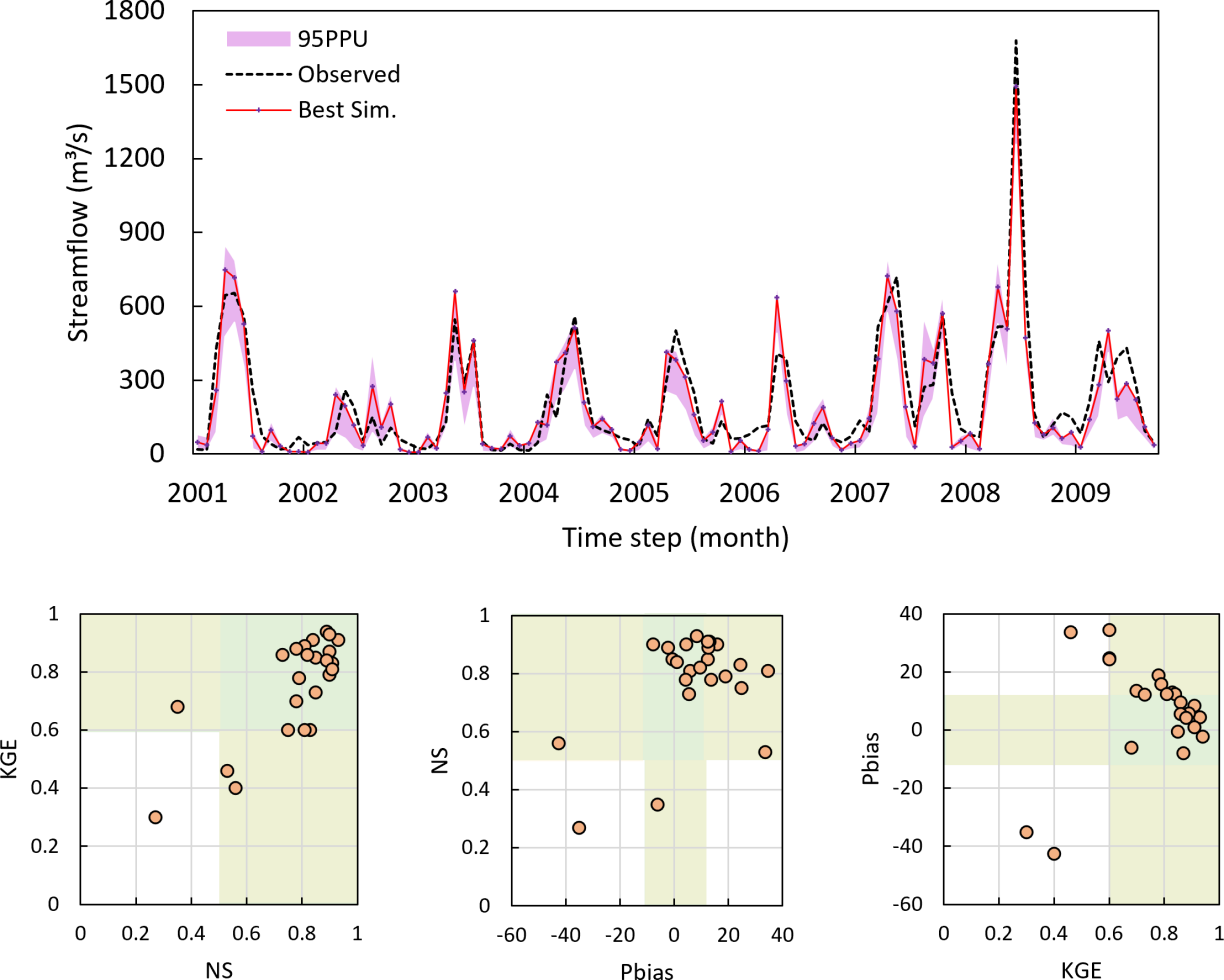
Best simulation results and uncertainty range for monthly streamflow calibration (hydrograph plot, see **Figure 1c** for gauge location) and spatial validation (dot plots) process represented by the 24 upstream monitoring stations. The shaded green area shows the acceptable range according Moriasi et al. (2015).

We assessed the baseline streamflow monthly calibration (2001 – 2009) and spatial validation process (2001–2010) (**Figure 4**). The calibration showed statistically acceptable performance, with an NSE of 0.85 and Pbias of 6.5%. The hydrograph suggests that the model performs well at predicting regular flows as well as seasonality. The validation results, with NSE values mostly > 0.50, and Pbias within ± 15% across all stations, further confirm an accurate model fit. The baseline nitrate simulations are generally consistent with measured levels and expected trends (average R² of 0.57 and Pbias of -23.5).

**Figure 5:**
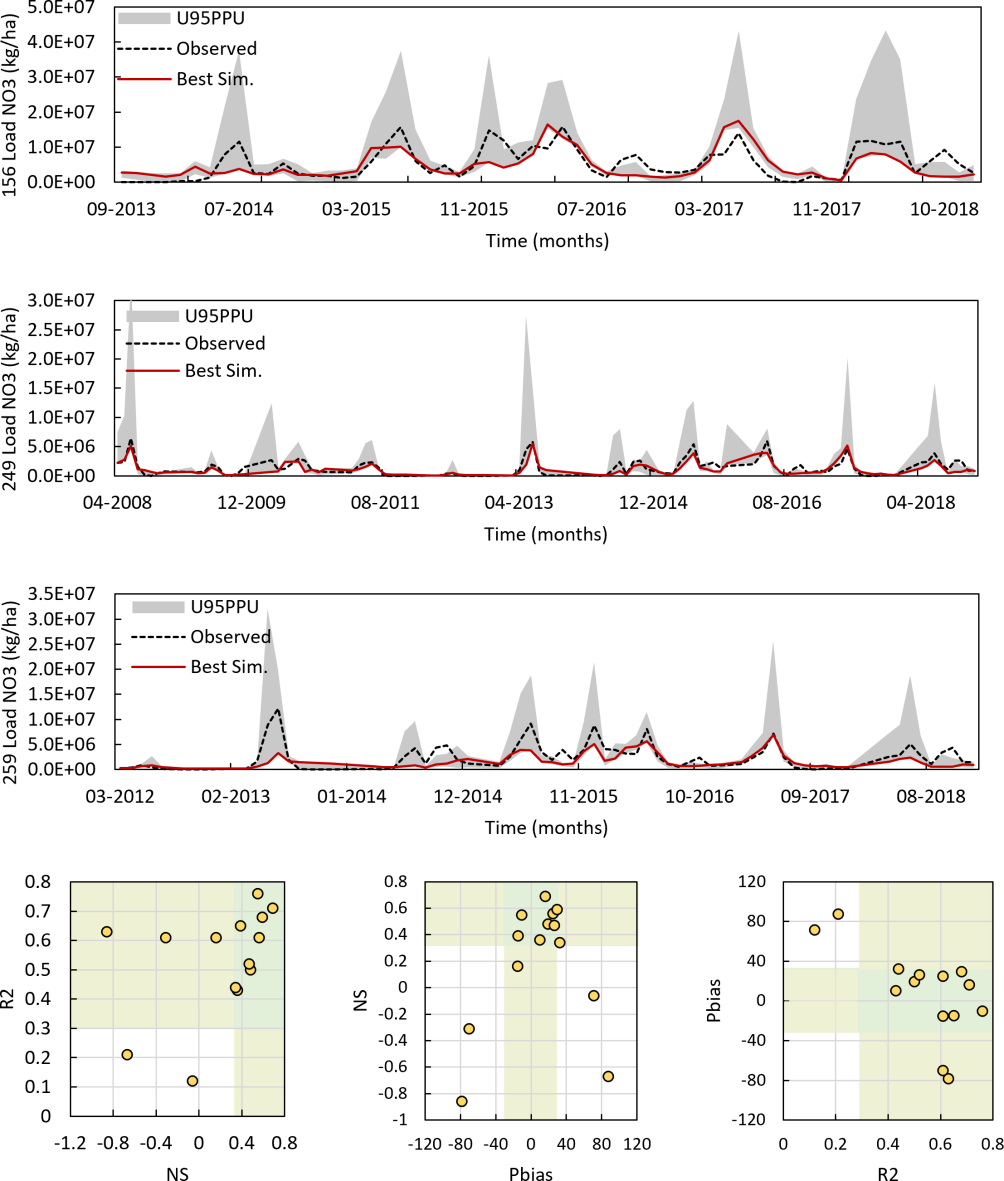
Best simulation results and uncertainty range for monthly nitrate calibration and observed data. The 3 time-series plots are representative of stations 156, 249, and 259, respectively (see **Figure 1** for gauge locations). The bottom dot plots are the statistical results for all 14 monitoring stations during the calibration process, the shaded green area shows the acceptable range according to Moriasi et al. (2015).

To improve NO_3_ simulation, we performed calibration using the best parameters from streamflow calibration and varied the nitrate parameters across 400 iterations (**Figure 5**). We then assessed simulated versus observed nitrate data from the 14 DMRB nitrate sensors (**Figure 1**).

Overall, the model underestimated nitrate levels at nine of the 14 stations, with underestimation of peak events (e.g., 156 and 259). The average correlation across stations was 0.55. The highest coefficient of determination was observed at station 190 (R² = 0.76), while the lowest was at station 358 (R² = 0.20). Model performance was moderate overall, with several sites showing satisfactory results (R² > 0.30, NSE > 0.35, and Pbias< ± 30), and seasonality was well captured (**Figure 5**).

The model’s reliability is supported by the uncertainty bounds (U95PPU) covering most of the observed data. However, the moderate correlation underscores considerable uncertainty regarding the model evaluation. Several factors may contribute to the uncertainty in management data. In this study, average planting, harvest, and fertilizer application dates representative of Iowa were applied uniformly across the watershed for row crops. While this approach reflects common production practices in the region, local variations may still occur. Fertilizer application rates were estimated using county-level census data and expert knowledge, which may affect the spatial distribution of inputs. Manure applications were also estimated due to the lack of detailed records, and manure was distributed evenly within subbasins containing livestock facilities, which may not fully represent actual management practices. In addition, nitrate transport was simulated using a simplified equation. These factors contribute to uncertainty in the nitrate simulations and should be considered when interpreting the results.

### 3.2 Final parameters and simulated yields

The final parameters implemented in the SWAT model for the 24 fruit and vegetable crops (**Table 5**) resulted in simulated yields that generally fell within a reasonable range of the expected dry yields. We examined simulated versus expected yields and the error associated with each crop simulation (**Table 6**). The Ep values, calculated to assess the model’s predictive performance for yield estimates, indicated that the majority of crops were simulated within the ±37% error threshold over the 13-year simulation period. The errors ranged from an underprediction of -25.9% for melons to an overprediction of 32.9% for onions. The model exhibited underpredictions for 12 of the crop types and overpredictions for the other 12 (**Table 6**).

**Table 5:** Final crop parameters used for SWAT model simulation (units and definition are included in **Supplementary Material**, **Supplementary Table 3**).

Parameter SWAT code	Parameter description	SCRN	POTA	SPOT	CRRT	ONIO	BROC	CABG	LETT
IDC	Land cover/plant classification	4	5	4	4	5	5	5	5
BIO_E	Radiation use efficiency	39	30	15	30	30	37.3	40	25
HVSTI	Harvest index	0.55	1.05	1.4	1.55	1.25	0.23	0.8	0.95
BLAI	Max Potential Leaf Area Index	3	5	5	3.1	1.5	4.83	3	4.62
FRGRW1	PHU fraction point 1	0.15	0.15	0.15	0.15	0.15	0.17	0.34	0.25
LAIMX1	BLAI fraction point 1	0.01	0.01	0.05	0.01	0.01	0.18	0.09	0.23
FRGRW2	PHU fraction point 2	0.5	0.5	0.5	0.5	0.5	0.67	0.78	0.4
LAIMX2	BLAI fraction point 2	0.95	0.95	0.95	0.95	0.95	0.62	0.99	0.86
DLAI	Growing season decline fraction	0.5	0.6	0.6	0.6	0.6	0.75	0.9	1
CHTMX	Maximum canopy height	2.5	1.2	0.8	0.3	1.2	0.5	0.33	0.2
RDMX	Maximum root depth	2	2	2	1.2	0.7	0.6	0.6	0.8
T_OPT	Optimal growth temperature	35	17	29	24	29	16	19	18.2
T_BASE	Minimum growth temperature	10	4.4	14	3.3	1.6	0	0	0
CNYLD	Nitrogen in yield	0.0214	0.0176	0.0097	0.0135	0.0206	0.0512	0.0259	0.0394
CPYLD	Phosphorus in yield	0.0037	0.0022	0.001	0.0036	0.0032	0.0071	0.0031	0.0049
PLTNFR (1)	Nitrogen uptake emergence	0.047	0.035	0.0532	0.055	0.04	0.062	0.062	0.05
PLTNFR (2)	Nitrogen uptake midseason	0.0177	0.02	0.02	0.0075	0.022	0.009	0.007	0.025
PLTNFR (3)	Nitrogen uptake maturity	0.0138	0.012	0.011	0.0012	0.02	0.007	0.004	0.021
PLTPFR (1)	Phosphorus uptake emergence	0.0048	0.006	0.0078	0.006	0.004	0.005	0.005	0.0084
PLTPFR (2)	Phosphorus uptake midseason	0.0018	0.0025	0.0033	0.003	0.002	0.004	0.0035	0.0032
PLTPFR (3)	Phosphorus uptake maturity	0.0014	0.0019	0.0015	0.002	0.0019	0.003	0.002	0.0019
WSYF	Minimum harvest index	0.28	1.05	1.4	1.12	1.25	0.2	0.8	0.95
USLE_C	Minimum C factor	0.2	0.2	0.05	0.2	0.2	0.2	0.2	0.01
GSI	Maximum stomatal conductance	0.007	0.007	0.01	0.006	0.007	0.006	0.006	0.0122
VPDFR	Vapor pressure deficit	4	4	4	4	4	4	4	4
FRGMAX	Max Stomatal Conductance 2	0.75	0.75	0.75	0.75	0.75	0.75	0.75	0.75
WAVP	BIO_E decline	0.5	10	1	10	10	5	5	8
CO2HI	Elevated CO2 concentration	660.45	660.41	660.2	660	660.41	660	660	660
BIOEHI	CO2HI biomass energy ratio	45	30	19	35	35	30	25	25
RSDCO_PL	Daily Residue Decomposition	0.05	0.05	0.05	0.05	0.05	0.05	0.05	0.05
ALAI_MIN	Minimum Leaf Area Index	0.5	2	1	0	0	0	0	0.1
BIO_LEAF	Biomass Fraction Leaf	0	0	0	0	0	0	0	0
MAT_YRS	Tree Years Maturity	0	0	0	0	0	0	0	0
BMX_TREES	Forest Maximum Biomass	0	0	0	0	0	0	0	0
EXT_COEF	Light Extinction Coefficient	0.65	0.65	0.65	0.65	0.65	0.65	0.65	0.65
BMDIEOFF	Biomass Dieoff Fraction	0.1	0.1	0.1	0.1	0.1	0.1	0.1	0.1

Parameter SWAT code	Parameter description	SPIN	CUCM	HMEL	STRW	TOMA	APPL	GRAP	BLUE
IDC	Land cover/plant classification	5	4	4	6	4	7	6	6
BIO_E	Radiation use efficiency	30	33	33	33	30	13.5	30	15
HVSTI	Harvest index	0.95	0.35	0.66	0.5	0.6	0.09	0.3	0.3
BLAI	Max Potential Leaf Area Index	4.2	1.5	4	3	3	4	2	1.5
FRGRW1	PHU fraction point 1	0.1	0.15	0.15	0.15	0.15	0.1	0.05	0.1
LAIMX1	BLAI fraction point 1	0.05	0.05	0.05	0.05	0.05	0.15	0.01	0.15
FRGRW2	PHU fraction point 2	0.9	0.5	0.5	0.5	0.5	0.5	0.5	0.5
LAIMX2	BLAI fraction point 2	0.95	0.95	0.95	0.95	0.95	0.75	0.95	0.75
DLAI	Growing season decline fraction	0.95	0.6	0.6	0.6	0.95	0.99	0.9	0.99
CHTMX	Maximum canopy height	1.2	0.5	0.8	0.8	0.8	3.5	2	1.5
RDMX	Maximum root depth	0.7	1.2	1.1	0.7	1.5	2	2	1.5
T_OPT	Optimal growth temperature	24	32	35	32	27	22	30	30
T_BASE	Minimum growth temperature	10	12.7	10	3.8	10	7	10	16
CNYLD	Nitrogen in yield	0.0544	0.0218	0.0072	0.0116	0.0235	0.0019	0.02	0.0019
CPYLD	Phosphorus in yield	0.0058	0.0043	0.001	0.0023	0.0038	0.0004	0.0025	0.0004
PLTNFR (1)	Nitrogen uptake emergence	0.07	0.04	0.02	0.0663	0.0663	0.006	0.01	0.006
PLTNFR (2)	Nitrogen uptake midseason	0.04	0.02	0.01	0.0255	0.03	0.002	0.004	0.002
PLTNFR (3)	Nitrogen uptake maturity	0.03	0.0015	0.008	0.0148	0.025	0.0015	0.003	0.0015
PLTPFR (1)	Phosphorus uptake emergence	0.005	0.0053	0.0026	0.0053	0.0053	0.0007	0.0014	0.0007
PLTPFR (2)	Phosphorus uptake midseason	0.004	0.0025	0.002	0.002	0.0035	0.0004	0.0008	0.0004
PLTPFR (3)	Phosphorus uptake maturity	0.0035	0.0012	0.0017	0.0012	0.0025	0.0003	0.0006	0.0003
WSYF	Minimum harvest index	0.95	0.35	0.55	0.5	0.33	0.09	0.3	0.3
USLE_C	Minimum C factor	0.2	0.03	0.03	0.03	0.03	0.001	0.1	0.1
GSI	Maximum stomatal conductance	0.01	0.007	0.007	0.007	0.007	0.007	0.005	0.007
VPDFR	Vapor pressure deficit	4	4	4	4	4	4	1.1	4
FRGMAX	Max Stomatal Conductance 2	0.75	0.75	0.75	0.75	0.75	0.75	0.75	0.75
WAVP	BIO_E decline	0.1	8	1	8	0.1	8	9	8
CO2HI	Elevated CO2 concentration	660.41	660	660.41	660.39	660.41	660	660	660.35
BIOEHI	CO2HI biomass energy ratio	35	39	39	39	39	20	40	40
RSDCO_PL	Daily Residue Decomposition	0.05	0.05	0.05	0.05	0.05	0.05	0.05	0.05
ALAI_MIN	Minimum Leaf Area Index	0.1	0	1	1	0.1	0.75	0.01	0
BIO_LEAF	Biomass Fraction Leaf	0	0	0	0	2	0.3	0.3	0.3
MAT_YRS	Tree Years Maturity	0	0	0	0	0	10	2	0
BMX_TREES	Forest Maximum Biomass	0	0	0	0	0	500	1	0
EXT_COEF	Light Extinction Coefficient	0.65	0.65	0.65	0.65	0.65	0.65	0.65	0.65
BMDIEOFF	Biomass Dieoff Fraction	0.1	0.1	0.1	0.1	0.1	0.1	0.1	0.1

Parameter SWAT code	Parameter description	CHER	COLG	DRYB	KALE	PEAR	PUMP	SQUA	RASP
IDC	Land cover/plant classification	7	5	1	5	7	4	4	6
BIO_E	Radiation use efficiency	15	23	25	23	13.9	30	25	30
HVSTI	Harvest index	0.1	0.8	0.4	0.8	0.09	0.5	0.64	0.2
BLAI	Max Potential Leaf Area Index	4	4.2	2	4.2	4	1.5	1.5	2
FRGRW1	PHU fraction point 1	0.1	0.25	0.58	0.25	0.1	0.15	0.15	0.05
LAIMX1	BLAI fraction point 1	0.15	0.23	0.05	0.23	0.15	0.01	0.05	0.01
FRGRW2	PHU fraction point 2	0.5	0.4	0.89	0.4	0.5	0.5	0.5	0.5
LAIMX2	BLAI fraction point 2	0.75	0.86	0.99	0.86	0.75	0.95	0.95	0.95
DLAI	Growing season decline fraction	0.99	1	0.75	1	0.99	0.6	0.75	0.9
CHTMX	Maximum canopy height	3.5	0.2	0.44	0.2	3.5	0.8	0.55	2
RDMX	Maximum root depth	2	0.8	1.1	0.8	2	1.1	1.1	2
T_OPT	Optimal growth temperature	28	18.2	30	18.2	35	35	25	30
T_BASE	Minimum growth temperature	7	4.4	8	0	10	10	7.5	10
CNYLD	Nitrogen in yield	0.0019	0.0394	0.0375	0.0394	0.0019	0.0117	0.0117	0.02
CPYLD	Phosphorus in yield	0.0004	0.0049	0.0047	0.0049	0.0004	0.0011	0.0011	0.0025
PLTNFR (1)	Nitrogen uptake emergence	0.006	0.05	0.045	0.05	0.006	0.025	0.025	0.01
PLTNFR (2)	Nitrogen uptake midseason	0.002	0.025	0.03	0.025	0.002	0.015	0.015	0.004
PLTNFR (3)	Nitrogen uptake maturity	0.0015	0.021	0.025	0.021	0.0015	0.01	0.01	0.003
PLTPFR (1)	Phosphorus uptake emergence	0.0007	0.0084	0.0045	0.0084	0.0007	0.0053	0.0053	0.0014
PLTPFR (2)	Phosphorus uptake midseason	0.0004	0.0032	0.003	0.0032	0.0004	0.0025	0.0025	0.0008
PLTPFR (3)	Phosphorus uptake maturity	0.0003	0.0019	0.0025	0.0019	0.0003	0.0012	0.0012	0.0006
WSYF	Minimum harvest index	0.1	0.8	0.3	0.8	0.09	0.25	0.25	0.02
USLE_C	Minimum C factor	0.001	0.01	0.2	0.01	0.001	0.03	0.03	0.1
GSI	Maximum stomatal conductance	0.007	0.0122	0.01	0.0122	0.007	0.007	0.007	0.005
VPDFR	Vapor pressure deficit	4	4	4	4	4	4	4	1.1
FRGMAX	Max Stomatal Conductance 2	0.75	0.75	0.75	0.75	0.75	0.75	0.75	0.75
WAVP	BIO_E decline	3	0.5	1	0.5	8	1	1	8
CO2HI	Elevated CO2 concentration	660	660.31	660	660.31	660	660.41	660.41	660
BIOEHI	CO2HI biomass energy ratio	20	25	27	25	20	39	39	40
RSDCO_PL	Daily Residue Decomposition	0.05	0.05	0.05	0.05	0.05	0.05	0.05	0.05
ALAI_MIN	Minimum Leaf Area Index	0.75	0	0	0	0.75	0	0	0.01
BIO_LEAF	Biomass Fraction Leaf	0.3	0	0	0	0.3	0	0	0.3
MAT_YRS	Tree Years Maturity	10	0	0	0	10	0	0	2
BMX_TREES	Forest Maximum Biomass	500	0	0	0	500	0	0	1
EXT_COEF	Light Extinction Coefficient	0.65	0.65	0.65	0.65	0.65	0.65	0.65	0.65
BMDIEOFF	Biomass Dieoff Fraction	0.1	0.1	0.1	0.1	0.1	0.1	0.1	0.1

^1^ The crop types presented are: SCRN (sweet corn), POTA (potato), SPOT (sweet potato), CRRT (carrots), ONIO (onions), BROC (broccoli), CABG (cabbage), LETT (lettuce), SPIN (spinach), CUCM (cucumber), HMEL (honeydew melon), STRW (strawberry), TOMA (tomatoes), APPL (apples), GRAP (grapes), BLUE (blueberries), CHER (cherries), COLG (collard greens), DRYB (dry beans), KALE (kale), PEAR (pear), PUMP (pumpkin), SQUA (squash), RASP (raspberry).

**Table 6:** Final simulated yields, expected yields, and the threshold error (Ep) associated with the simulations.

Crops	Yield (ton/ha)		Ep (%)	Crops	Yield (ton/ha)		Ep (%)
	Expected	Simulated			Expected	Simulated	
Apple	5.2	6.7	29.3	Lettuce	2.2	1.6	-25.6
Blueberries	1.2	1.0	-16.6	Melon	3.0	2.2	-25.9
Broccoli	1.6	2.0	24.6	Onions	5.4	7.1	32.9
Cabbage	4.4	4.0	-8.9	Pears	6.0	5.9	-0.1
Carrots	7.5	9.7	29.6	Potatoes	9.5	7.3	-23.6
Cherries	7.1	8.6	21.2	Pumpkin	2.9	2.2	-24.8
Collard greens	0.9	1.2	33.1	Raspberries	1.1	1.8	8.1
Sweet corn	4.6	5.8	26.1	Spinach	1.5	1.8	21.3
Cucumber	0.8	0.7	-12.3	Squash	2.1	2.6	23.5
Dry beans	1.7	1.4	-21.1	Strawberries	4.7	4.5	-3.7
Grapes	3.9	3.3	-14.1	Sweet potatoes	6.0	5.1	-13.8
Kale	4.6	5.7	21.2	Tomatoes	5.4	6.8	26.3

## 4 Discussion

### 4.1 Improved SWAT models simulation

Nitrate characteristics at the watershed scale are highly interactive with the hydrological cycle, especially in areas with extensive tile drainage, like the DMRB. The tiles act as conduits in these regions, rapidly transporting nitrate to stream networks (Gassman et al., 2017; Schilling et al., 2019). We assume the methodological approach gives accurate quantification of nutrient applications from manure and commercial fertilizer, calibration results, and given the model’s strong calibration fit for tile drainage. Future work will focus on reducing uncertainty by refining parameter types and ranges, and by reevaluating manure distribution, including the potential for manure transport between subbasins over maximum distances (km) depending on manure type.

One of the major challenges we face was that the availability of data for fruit and vegetable crops (and their corresponding growth parameters) was inconsistent across the literature, other models (EPIC, APEX, ALMANAC), and expert estimates. Ensuring the same quality of information for 24 crops across 37 parameters each is challenging. It is not always possible to follow the same modification steps for all crops. For example, no direct data was available for pears, so several assumptions had to be made based on similarities with apples. In contrast, broccoli had much more experimental data available, describing 11 parameters, which reduces uncertainty associated with the results.

The collected information was used to construct a counts matrix, where each data point corresponds to a different source. The matrix includes all crops and all parameters, showing whether data are available for a given crop–parameter combination and how many sources provide that information (**Appendix: Table A1**). For example, a value of “6” in the BIO_E column for apple indicates that six different sources contain information for that parameter: the SWAT, APEX, and EPIC databases, two scientific papers and one book chapter; additionally, we can identify which parameters are the most common: BIO_E was present in six sources, HVSTI in five sources, and BLAI in three sources.

In general, the T_BASE and HVSTI parameters were the most frequently reported, with 98 and 97 data points across all crops, followed by T_OPT, BIO_E, and RDMX, with 89, 85, and 81, respectively. In contrast, values for parameters such as GSI, FRGMAX, and VPDFR were less common, with values obtained exclusively from the SWAT, EPIC, and APEX databases. These three specific parameters are used in the SWAT simulations when the Penman-Monteith method for calculation of maximum plant evapotranspiration is selected. The respective plant databases include these variables because they are related to stomatal conductance, which are necessary only when the Penman-Monteith equations are used: maximum stomatal conductance (GSI), and two variables that describe how vapor pressure deficit influences stomatal conductance (FRGMAX and VPDFR).

Among the 24 crops, lettuce, grapes, and potatoes were the most well-represented for many parameters, reflecting multiple data sources. In contrast, crops like pumpkin, kale, and collard greens show consistently low counts (often limited to a single source), indicating data scarcity and reliance on extrapolations from related crops as well as expert estimates (**Table 3**). This data limitation is particularly relevant for Penman–Monteith simulations, because crop controls on evapotranspiration in SWAT are influenced by both canopy development/growth parameters (e.g., LAI and phenology controls in plant.dat) and the Penman–Monteith-specific stomatal conductance response variables (GSI, FRGMAX, VPDFR). In this study, these Penman–Monteith-specific parameters were not independently calibrated against crop ET observations (which are not available for most specialty crops in the study region) and were therefore applied as part of the assembled crop parameter sets from SWAT/EPIC/APEX sources. We acknowledge that potential inconsistencies between canopy-development parameters and Penman–Monteith stomatal controls may affect simulated ET and the timing/magnitude of water stress for some crops, and this represents an important uncertainty for future work. These variations in crop information and Pbias reflect the challenges of accurately representing diverse crop growth patterns within the SWAT framework, especially for under-represented table food crops with limited regional data.

**Figure 6:**
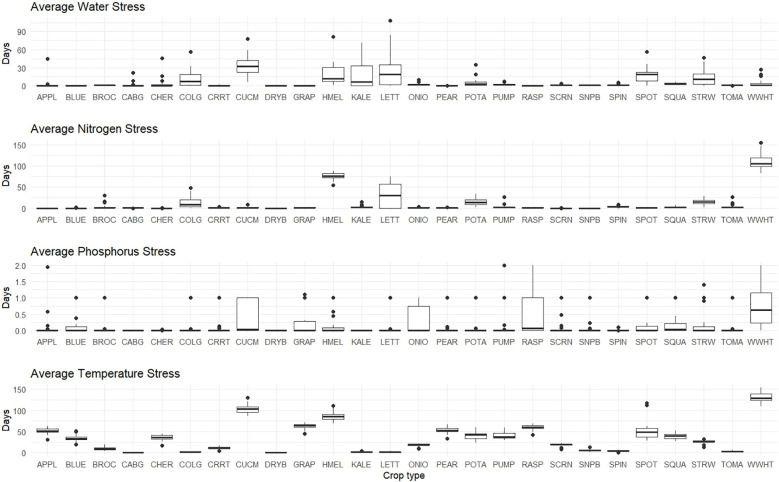
Average number of accumulated days under stress for each crop (water, nitrogen, phosphorus, and temperature).

To better document the extent to which the adopted management assumptions (no irrigation and SWAT auto-fertilization) resulted in stress-free growth, we evaluated SWAT plant stress diagnostics for all crops and summarized them as mean annual stress days (i.e., the average number of accumulated days under stress per year) for water, nitrogen, phosphorus, and temperature (**Figure 6**). This diagnostic indicates that most crops experienced limited water and nutrient stress under the simulated conditions; however, meaningful water stress occurred for several horticultural crops, particularly collard greens, cucumbers, kale, lettuce, and honeydew melon (**Figure 6**). These results clarify that the “no irrigation” assumption does not imply “no water stress” for all crops, especially shallow-rooted vegetables. Consequently, for crops that are irrigated in practice, simulated yields and nutrient uptake may be biased low relative to irrigated management, and our results for these crops should be interpreted primarily as rainfed outcomes for the modeled soils and climate. In contrast, the use of SWAT auto-fertilization tends to reduce nutrient limitation, which may bias yields and nutrient uptake upward relative to producer-managed nutrient constraints.

### 4.2 Insight into data application

The fruit and vegetable crop parameters presented in this study hold potential for applications in the Corn Belt and similar regions. Here, the SWAT model was used to assess crop yields under historical and future climate scenarios and potential plant uptake of nutrients. Precipitation and temperature projections were obtained through the CORDEX initiative (https://cordex.org/) using a Regional Climate Model (RCM) and a Global Climate Model (GCM). Specifically, we used the WRF-GFDL-ESM2M climate model combination to simulate yields for historical and future conditions. The GCM-RCM combination was selected based on results from a previous study conducted at the same site (Brighenti et al., 2023b). For this experiment, the model was run from 1983 to 2050.

**Figure 7:**
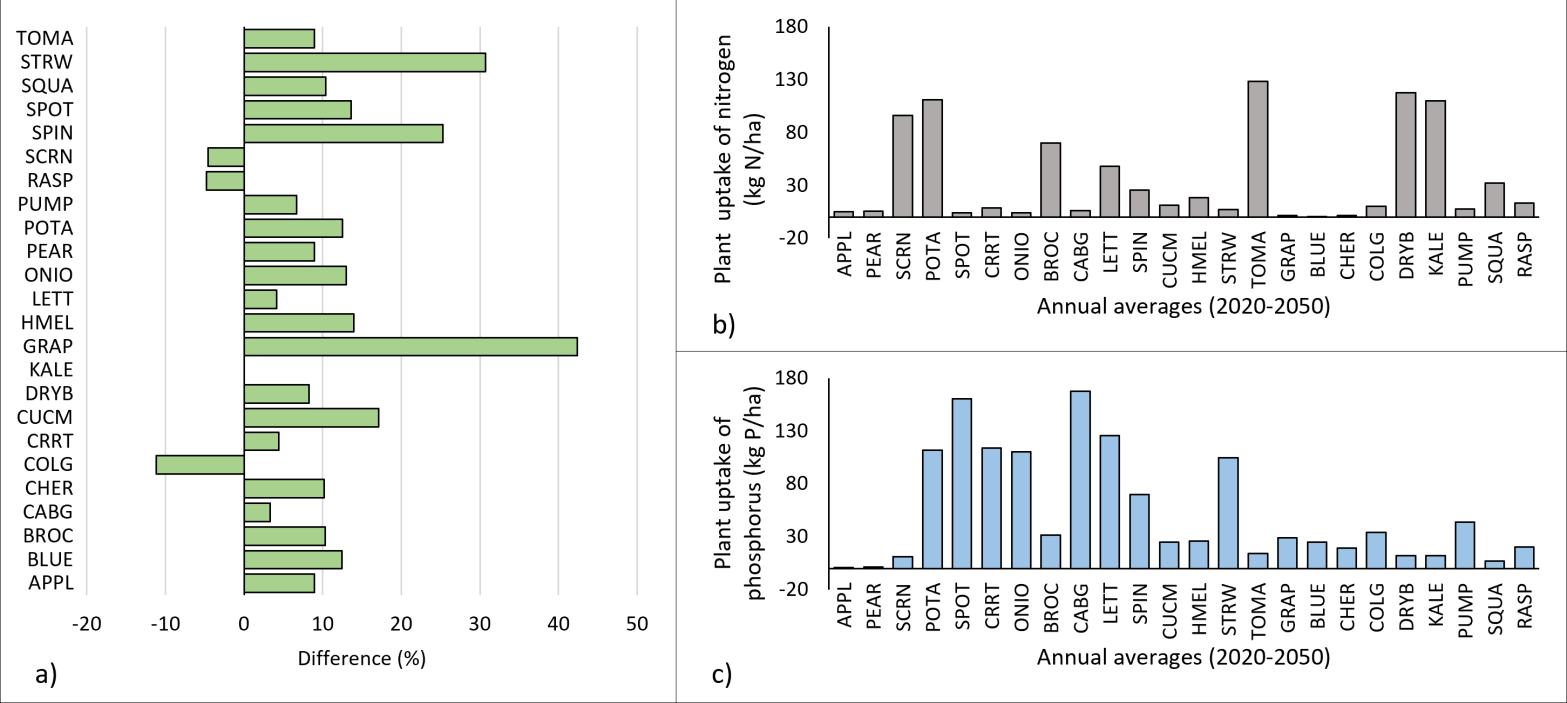
**(A)** Difference (%) of crop yield from the historical run (1983-2005) comparing to the future projection (2039-2050), **(B)** Plant uptake of nitrogen (kg N/ha), and **(C)** plant uptake of phosphorus (kg N/ha).

Our findings indicate that changes in future precipitation and temperature patterns could lead to varying crop yield outcomes. We assessed the percentage differences in crop yields between the historical simulation (1983-2005) and future projection (2039-2050), and the plant uptake of nitrogen and phosphorus (**Figure 7**). Most crops show an increase in projected yield relative to the historical baseline except for collard greens, raspberry, and sweet corn. Overall, this analysis highlights variability among crops (**Figure 7a**), with some producing higher yields under projected future climate conditions. These differences in yield response can be partially related to crop growth characteristics represented in the model, including parameters such as T_BASE and T_OPT, for example. Crops with temperature thresholds that align more closely with the projected climate conditions may experience improved growth, while crops that are more sensitive to heat or water stress may show reduced yields under the same conditions.

Regarding nutrient removal from the soil by plants, the SWAT model monitors both nitrogen and phosphorus dynamics. We determined the maximum annual uptake per hectare, representing the nutrient absorption potential of each crop variety. The uptake of these nutrients varies among crop types and is different between nitrogen and phosphorus (**Figures 7b, c**). Fertilization in the model was applied automatically, ensuring that each crop received the optimal nutrient supply for maximum growth. For example, crops such as potatoes have high uptake rates for both nitrogen and phosphorus. Such approaches (variations in nutrient demand and potential increase or decrease in yields) can be used in agricultural planning and decision-making in response to anticipated effects of climate change.

## 5 Conclusion

This study provides a comprehensive framework demonstrating the ability of the SWAT model to simulate fruit and vegetable crop yields in a region with limited data availability for these specific crops. It addresses gaps in the model for table foods by combining data from the SWAT, EPIC and APEX models, expert opinion, literature, and expected and simulated yield comparisons.

The SWAT simulations were characterized by an error margin for simulated yields of ±37%, which is acceptable due to the region’s limited yield data for fruits and vegetables. Of the 24 crops analyzed, 15 were present in the SWAT database and revised using EPIC/APEX crop parameters, literature review, crop calendars, and expert guidance. The parameters for the nine additional crops were developed using a similar process The crop parameters established in this study will support SWAT simulations for the larger regional project and other ongoing research initiatives such as the Diverse Corn Belt Project (https://diversecornbelt.org/) and provide insights for development of localized food production strategies.

To share these findings with the broader scientific community, we are planning to insert the adjusted and new parameter sets into the default SWAT2012 database and encourage modelers to use them in their regional studies, making informed adjustments as needed. These parameters are optimized for Western Corn Belt conditions; thus, their extrapolation to other regions will require careful consideration of regional climate, soil, and crop management practices. Users are encouraged to consult relevant literature and conduct additional testing to ensure the reliable application of these parameters elsewhere. The proposed parameter integration methodology (**Figure 3**) can also be replicated for other underrepresented crop types in SWAT or in the next generation SWAT+ model.

The model analysis for streamflow and nitrate confirmed reliable simulations of hydrological processes and nutrient transport. Calibration and validation results indicate strong model performance, with metrics such as NSE (0.85) and Pbias (6.5%) confirming accuracy in reproducing streamflow patterns and seasonality. The current model replication of in-stream nitrate levels revealed moderate model performance, with several sites showing satisfactory fit (R² > 0.30, NSE > 0.35, Pbias< ± 30). Climate projections using the SWAT model suggest crop yield variability in response to future scenarios. Crops like strawberries, cucumbers, and broccoli may produce higher yields given predicted climate shifts, while sweet corn, raspberries, and collard greens will likely experience yield declines. These results highlight the importance of targeted agricultural planning to adapt to changing environmental conditions.

Future research should evaluate the crop parameters across different climates, examine irrigation practices for crops like tomatoes, lettuce, and broccoli, and rely on longer-term yield datasets. Further work in Iowa should focus on optimizing water use and collecting measured yield data for the crops analyzed to refine simulations and expand the utility of SWAT models. Also, further refinement in nitrate-specific parameters and input data on nutrient applications is recommended to improve simulation accuracy. The framework established in this study provides a basis for sustainable agricultural planning and climate resilience, particularly for localized production of table food crops.

## Data Availability

The original contributions presented in the study are included in the article/**Supplementary Material**. Further inquiries can be directed to the corresponding author.

## Appendix A

**Table A1:** Counts matrix showing the number of independent sources available for 37 crop growth parameters for 24 fruit and vegetable crops. Rows correspond to individual crops and columns headings to parameters.

	BIO-E	HVSTI	BLAI	FRGRW1	LAIMX1	FRGRW2	LAIMX2	DLAI	CHTMX	RDMX	T-OPT	T-BASE	CNYLD	CPYLD	PLTNFR(1)	PLTNFR(2)	PLTNFR(3)	PLTPFR(1)	PLTPFR(2)	PLTPFR(3)	WSYF	USLE-C	GSI	VPDFR	FRGMAX	WAVP	CO2HI	BIOEHI	RSDCO-PL	ALAI_MIN	BIO-LEAF	MAT-YRS	BMX-TREES	EXT-COEF	BMDIEOFF
APPL	6	5	4	3	3	3	3	4	5	4	5	6	3	3	3	3	3	3	3	3	3	1	3	1	1	4	3	1	1	4	1	1	1	1	1
BLUE	1	2	1	1	1	1	1	1	1	1	2	2	1	1	1	1	1	1	1	1	2	1	1	1	0	2	1	0	0	1	0	0	0	0	0
BROC	4	5	4	4	4	4	4	4	3	4	5	5	3	3	3	3	3	3	3	3	4	1	3	1	1	3	3	1	1	3	1	1	1	1	1
CABG	4	8	3	4	4	4	4	4	4	6	7	7	3	3	3	3	3	3	3	3	3	1	3	1	1	3	3	1	1	3	1	1	1	1	1
CRRT	4	8	4	3	1	3	1	3	3	5	5	6	3	3	3	3	3	3	3	3	4	1	3	1	1	3	3	1	1	3	1	1	1	1	1
CHER	4	3	3	3	3	3	3	3	4	4	5	5	3	3	3	3	3	3	3	3	3	1	3	1	1	3	3	1	1	3	1	1	1	1	1
COLG	1	1	1	1	1	1	1	1	1	1	1	1	1	1	1	1	1	1	1	1	2	0	1	0	0	1	1	0	0	1	0	0	0	0	0
CUCM	4	4	3	3	3	3	3	3	3	3	3	3	3	3	3	3	3	3	3	3	4	1	3	1	1	3	3	1	1	3	1	1	1	1	1
DRYB	5	5	4	4	4	4	4	4	5	4	5	5	2	2	2	2	2	2	2	2	2	0	2	0	0	2	2	0	2	2	0	0	0	1	0
GRAP	6	6	5	5	5	5	5	5	6	6	6	6	5	3	3	3	5	3	3	5	5	2	5	2	0	5	3	0	3	5	0	2	0	0	0
LETT	6	5	6	5	1	5	1	5	5	5	5	5	5	5	5	5	5	5	5	5	6	1	5	1	1	5	5	1	1	5	1	1	1	1	1
KALE	1	1	1	1	1	1	1	1	1	1	1	1	1	1	1	1	1	1	1	1	1	0	1	0	0	1	1	0	0	1	0	0	0	0	0
HMEL	4	7	4	3	3	3	3	3	3	4	4	5	3	3	3	3	3	3	3	3	4	1	3	1	1	3	3	1	1	4	1	1	1	1	1
ONIO	3	4	3	3	1	3	1	3	3	4	4	5	3	3	3	3	3	3	3	3	4	1	3	1	1	3	3	1	1	3	1	1	1	1	1
PEAR	6	4	4	3	3	3	3	3	4	5	6	6	3	3	3	3	3	3	3	3	3	1	3	1	1	3	3	1	1	3	1	1	1	2	1
POTA	4	5	4	4	4	4	4	4	4	4	4	5	4	4	4	4	4	4	4	4	4	2	3	1	1	4	3	1	1	4	1	1	1	1	1
PUMP	1	1	1	1	1	1	1	1	1	1	1	1	1	1	1	1	1	1	1	1	1	0	1	0	0	1	1	0	0	1	0	0	0	0	0
RASP	2	2	1	1	1	1	1	1	2	2	2	2	1	1	1	1	1	1	1	1	1	0	1	0	0	1	1	0	1	1	0	0	0	0	0
SPIN	3	3	3	3	1	3	1	3	3	3	3	4	3	3	3	3	3	3	3	3	3	1	3	1	1	3	3	1	1	3	1	1	1	1	1
SQUA	2	2	1	2	2	2	2	2	2	1	2	3	1	1	1	1	1	1	1	1	1	0	1	0	0	1	1	0	0	1	0	0	0	0	0
STRW	4	5	3	3	3	3	3	3	3	3	3	4	3	3	3	3	3	3	3	3	3	1	3	1	1	3	3	1	1	3	1	1	1	1	1
SCRN	3	3	3	3	2	3	1	3	3	3	3	4	3	3	3	3	3	3	3	3	3	1	3	1	1	3	3	1	1	3	1	1	1	1	1
SPOT	3	4	3	3	2	3	1	3	3	3	3	3	3	3	3	3	3	3	3	3	3	1	3	1	1	3	3	1	1	3	1	1	1	1	1
TOMA	4	4	4	4	4	4	4	4	4	4	4	4	4	4	4	4	4	4	4	4	3	1	4	1	1	4	3	1	1	4	3	1	1	1	1

Rows correspond to individual crops and columns to parameters.
