# Supplementary material for: Integrating new fruit and vegetable growth parameters in SWAT models for improved simulations
Source: Front Plant Sci. 2026 May 26;17:1745017. doi: 10.3389/fpls.2026.1745017 (PMC13248021; doi:10.3389/fpls.2026.1745017)
Supplement: Supplementary file 1 [file SupplementaryFile1.pdf]

Supplementary Material

Integrating New Fruit and Vegetable Growth Parameters in SWAT Models for Improved Simulations

Table S1. Additional studies that report simulation of fruit and/or vegetable crops but did not report simulated versus observed yield comparisons or any calibrated crop parameters.

Source	Watershed (km^2); region	Specific fruit and vegetable crops simulated [1]	Type of analysis
Heidari et al., (2020)	Mun River (71,060); northeast Thailand	cassava, orchard	climate change and land use change impacts on water balance
Bannwarth et al., (2016)	Mae Sa (77); northwest Thailand	tomato, bell pepper, onion, lettuce, Chinese cabbage, green bean, white cabbage, cayote, litchi	pesticide impacts on surface water
Jordan et al., (2018)	Bulgan River (8,015); western Mongolia	melon, potato, sea buckthorn, fruit tree, vegetable[2]	assessment of water use in agro-pastoral production systems
Msigwa et al., (2022) [3]	Kikuletwa River (6,650); northeast Tazania	banana, tomato, onion, bean	seasonal land use impacts on blue and green water consumption
Nkwasa et al., (2020) [3]	Usa River (240); northeast Tazania	banana, tomato, eggplant	seasonal land use effects on blue and green water use
Nigussie et al., (2019)	Jabitenan District (12,000); Blue Nile River Basin, northwest Ethiopia	pepper, tomatoes, onions, potatoes, other vegetables [2]	surface irrigation assessment for several different cropping systems
Dawit et al., (2020)	Dhidhessa River (14,710); northwest Ethiopia	cabbage, tomato, pepper, groundnut	surface irrigation assessment for several different cropping systems
Amon-Armah et al., (2013; Li et al., (2014)	Black Brook (14.5); northeast New Brunswick, Canada	potato, carrot, field pea	nutrient management, crop yields, recharge rate, nitrogen leaching, and/or sediment loss
Garg et al., (2021)	“fragile watershed” (48); Bundi District; Rajasthan State, western India	chickpea, pigeon pea	rainwater harvesting impacts on water resources

Jamshidi and Naderi (2023)	Zrebar Lake Basin (90); western Iran	grape, peas, tomato, apple	BMP impacts on pollution loads, crop yields, and water footprints
Bulut and Aksoy (2008)	Mustafakemalpasa River (813); northwest Turkey	tomato	fertilization & cropping system impacts on phosphorus transport
Huang et al., (2014)	Beijing & Shouguang Regions, northeast China[4]	tomato	crop water footprint assessment
Zhang et al., (2014)	Lizixi River (697.5); central China	sweet potato	land use change impacts on soil erosion
Mehdi et al., (2015)	Altmühl River (980); State of Bavaria, Germany	potato, strawberry, sugar beet, orchard [2]	impacts of climate & land use change on streamflow, & nitrate & phosphorus loss
Babel et al., (2021)	Huai Bang Sai River (1,340); northeast Thailand	cassava	assessment of the effectiveness of ecosystem-based adaptation measures such as reforestation and filter strips
Mararakanye et al., (2022)	Vaal River (7,220); north central South Africa	potato, onion	evaluation of long-term land use changes on water quality
Martínez-asasnovas et al., (2016)	Alt Penedès–Anoia Region (323.6); Catalonia, Spain	grape	soil loss calculations for a vineyard production region
Nkwasa et al., (2022) [3]	Northeast Africa (4,489,000); Nile Basin countries	banana	improved representation of crop phenology for rainfed and irrigated cropping systems in northeast Africa
Zorratipour et al., (2021)	Bakhtegan Lake[h] (3,227.3); southwest Iran	tomato, almond, apple	SWAT hydrologic testing for extensively tile-drained watershed
Jalali et al., (2021)	Zayandehrud River (917); central Iran	sugar beet, cucurbits [2], potato	impacts of climate change and human activities on runoff
Liang et al., (2020)	Wilmot River (45.6); west-central Prince Edward Island, Canada	potato	water yield, nitrate load
Yiridoe et al., (2017)	Thomas Brook (7.6): Kings County, Nova Scotia, Canada	potato, carrot	fertilizer rates, nitrate leaching
Pan et al., (2017)	Beijiang River (39,220); southeast China	orchard [2]	hydrologic impacts of climate and land use change

Zou et al., (2018)	Upper/Middle Heihe River (35,609); northwest China	Vegetables [2]	regional irrigation water demand
Zhang et al., (2023)	Middle Heihe River; northwest China	Vegetables [2], potato	climate change impacts on irrigation demand & optimal crop production
Han et al., (2023)	Wulong River (2,740); Shandong Province, China	apple	effect of nitrogen and phosphorus fertilization rates on nutrient transport
Eini et al., (2023)	Welna River; Wielkopolskie Province, Poland	potato, sugar beet	climate change impacts on crop yields
Halecki et al., (2018)	Mątny Mountain Basin; Małopolskie Province, Poland	potato	land use impacts on sediment loss
Piniewski et al., (2020)	Vistula & Odra Rivers; majority of Poland	potato	historical and future climate impacts on soil moisture levels and crop production
Curk et al., (2020)	Krška Kotlina Alluvial Plain (70); southeast Slovenia	bell pepper, potato, orchard [2]	nitrate pollution pathways as a function of cropping system
Donmez et al., (2020)	Lower Seyhan Plain (2,132); south central Turkey	citrus, peanut, vegetables [2]	hydrologic response and nitrate loss as a function of cropping system
Venishetty and Parajuli (2022)	Yazoo River (50,800); northwest Mississippi, United States	sweet potato	BMP impacts on hydrologic and water quality responses
Gunacti et al., (2022)	Küçük Menderes River (3,454); south of İzmir, Turkey	olives [5]	effects of land use changes on watershed water balance
Gholami et al., (2022)	Zarrineh River (2,532.5); northwest Iran	potato, tomato, sugar beet, apple	sensitivity assessment of SWAT output in response to different digital elevation models (DEMs)
Huo et al., (2020)	Huai Bang Sai River (1,340); east central Thailand	cassava	hydrologic simulation comparison between the SWAT and HEC-HMS models
Marcinkowski et al., (2023)	Vistula, Odra and other Rivers (386,166); entire country of Poland [6]	potato, sugar beet, vegetables	development of long-term simulated water balance and streamflow data for Poland

Gholami et al., (2023)	Chalus River (1,616); north central Iran	gardens [7]	land use change impacts on watershed hydrology
dos Santos et al., (2023)	Mogi Guaçu River (17,303); Sao Paulo State [8], southeast Brazil	beans, potatoes, citrus	determination of erosive areas and sediment transport
Razi and Shourian (2022)	Minab Reservoir Basin (10,313); western Iran	watermelon, cucumber	optimization was performed to maximize revenue and minimize nutrient loss

†[1] It is assumed that explicit crops listed in these studies were actually simulated by the authors, unless otherwise stated, but this cannot be absolutely verified for every study; [2] specific crops not reported for these generic crop categories; [3] used the SWAT+ model (https://swat.tamu.edu/software/plus/); [4] SWAT results were based on outputs reported in a previous study; [5] olive trees, orchards, vineyards and similar crops were simulated as olives; [6] drainage area includes a total of 37,087 km located in Ukraine, Belarus, Czech Republic, Germany, Slovakia or Russia; [7] no information was provided regarding crops that were included within the general gardens land use; [8] the majority of the watershed drainage area was in Sao Paulo State.

Table S2. Previous studies reporting comparison of fruit and vegetable yields estimated with SWAT versus corresponding expected yields, and/or calibration of two or more plant parameters required for simulating specific fruit and vegetable crops in SWAT.

Source	Watershed (km^2); region	Total fruit/ vegetable crop types	SWAT simulated yields vs. observed crop yields? [1]	Total crop parameters reported	Specific fruit and vegetable crops simulated
Akhavan et al., (2010)	Hamadan-Bahar River (2,460); Iran	1	yes	2	potato
Napoli and Orlandini (2015) [2]	Two fields (1.9 & 1.4 ha); Tuscany, Italy	2	no [3]	27 [4]	grape, olive
Wabela et al., (2022)	Bilate River (5,223); Ethiopia	1	Yes	7	potato
Cakir et al., (2020)	Garonne River (50,000); France	5	yes (3 crops [5])	3 [6]	olive, grape, fruit trees [7], berry [7], almond
Delavar et al., (2022) [2]	Karkheh River (42,267); Iran	3	yes [8]	10	sugar beet, tomato, apple
Emami and Koch (2018)	Zarrine River (12,000); northwest Iran	4	yes	11	tomato, potato, sugar beet, apple
Jeyrani et al., (2021)	Tashk-Bakhtegan Lakes (27,500); Iran	4	yes	none	almond, bean, grape, fig
Goudarzi et al., (2019)	Bakhtegan-Maharlu Lakes (1,750); Iran	5	yes	11	almond, apple, peach, tomato, olive
Paul et al., (2021) [2]	San Joaquin River (15,358); California, U.S.	2	yes	9 [6]	almond, grape
Raij-Hoffman et al., (2022)	Southern San Joaquin Valley, California, U.S.	1	yes	16	tomato
Xue et al., (2021)	San Joaquin River (15,000); California, U.S.	5	yes (4 crops)	12	almond, pistachio, walnut, grape, tomato
Samimi et al., (2022)	Middle Rio Grande River (6,000); New Mexico, U.S.	4 [9]	no	3	pecan; lettuce, onion, pepper
Udias et al., (2018)	Island of Crete (8,336); Greece	14	yes	none	olive, grape, potato, citrus [7], tomato, almond, carrot, onion, watermelon, green bean, cucumber, fruits [7], cabbage, eggplant
Liang et al., (2019)	Haean River (67.2); Kang-won Province, South Korea	3	no	8 [6]	cabbage, potato, radish
Kang et al., (2022)	Black Brook (14.5); New Brunswick, Canada	1	yes	none	potato

Igder et al., (2022) [10]	Simin-Dasht River (2,500); northern Iran	2	no	7	potato, orchard [7]
amshidi et al (2022)	Zrebar Lake Basin (90); western Iran	4	no	11	grape, apple, tomato, pea
Yousefi and Moridi (2022)	Minab Reservoir Basin (10,313); southern Iran	2	yes	10	eggplant, lima bean

†[1] ”yes” indicates studies that reported SWAT simulated crop yields versus corresponding observed crop yields; [2] modified SWAT model was used; [3] comparisons between simulated and observed above-ground biomass, dry matter and other plant indicators reported; [4] USLE_C reported as a plant parameter; [5] simulated versus observed crop yields reported for almond, fruit trees and grape; [6] adjusted plant parameters reported in corresponding supplementary documentation; [7] specific crops not reported for these generic crop categories; [8] simulated versus observed crop yields reported on the basis of garden, tuberous crops and other general crop categories; [9] authors state that they simulated pecan, alfalfa, corn, cotton, and vegetables, but explicitly list lettuce, onion, pepper and pecan in Figure 3; thus, it is assumed that they simulated those four crops in their study; [10] the authors report using the SWAT+ model.

Table S3 – Description of the crop growth parameters used for SWAT model simulations.

Acronym	Parameters	Description - Unit
BIO_E	Radiation use efficiency	Radiation-use efficiency (RUE) is the amount of dry biomass produced per unit intercepted solar radiation. The radiation-use efficiency is assumed to be independent of the plant's growth stage. BIO_E represents the potential or unstressed growth rate (including roots) per unit of intercepted photosynthetically active radiation. (kg/ha)/(MJ/m2)
HVSTI	Harvest index	The harvest index defines the fraction of the aboveground biomass that is removed in a harvest operation. This value defines the fraction of plant biomass that is "lost" from the system and unavailable for conversion to residue and subsequent decomposition. no unit
BLAI	Max Potential Leaf Area Index	BLAI is one of six parameters use to quantify leaf area development of a plant species during the growing season. no unit
FRGRW1	PHU fraction point 1	Fraction of the plant growing season or fraction of total potential heat units corresponding to the 1st point on the optimal leaf area development curve. %
LAIMX1	BLAI fraction point 1	Fraction of the maximum leaf area index corresponding to the 1st point on the optimal leaf area development curve. %
FRGRW2	PHU fraction point 2	Fraction of the plant growing season or fraction of total potential heat units corresponding to the 2nd point on the optimal leaf area development curve. %
LAIMX2	BLAI fraction point 2	Fraction of the maximum leaf area index corresponding to the 2nd point on the optimal leaf area development curve. %
DLAI	Growing season decline fraction	Fraction of growing season when leaf area begins to decline. no unit
CHTMX	Maximum canopy height	Maximum canopy height is a straightforward measurement. (m)
RDMX	Maximum root depth	Maximum root depth is a straightforward measurement. (m)
T_OPT	Optimal growth temperature	Both optimal and base temperatures are very stable for cultivars within a species. (ºC)
T_BASE	Minimum growth temperature	Minimum (base) temperature for plant growth (ºC).
CNYLD	Nitrogen in yield	Normal fraction of nitrogen in yield. (kg N/kg yield)
CPYLD	Phosphorus in yield	Normal fraction of phosphorus in yield. (kg P/kg yield)
PLTNFR (1)	Nitrogen uptake emergence	Nitrogen uptake parameter #1: normal fraction of nitrogen in plant biomass at emergence. (kg N/kg biomass)
PLTNFR (2)	Nitrogen uptake midseason	Nitrogen uptake parameter #2: normal fraction of nitrogen in plant biomass at 50% maturity. (kg N/kg biomass)
PLTNFR (3)	Nitrogen uptake maturity	Nitrogen uptake parameter #3: normal fraction of nitrogen in plant biomass at maturity. (kg N/kg biomass)
PLTPFR (1)	Phosphorus uptake emergence	Phosphorus uptake parameter #1: normal fraction of phosphorus in plant biomass at emergence. (kg P/kg biomass)
PLTPFR (2)	Phosphorus uptake midseason	Phosphorus uptake parameter #2: normal fraction of phosphorus in plant biomass at 50% maturity. (kg P/kg biomass)
PLTPFR (3)	Phosphorus uptake maturity	Phosphorus uptake parameter #3: normal fraction of phosphorus in plant biomass at maturity. (kg P/kg biomass)
WSYF	Minimum harvest index	Lower limit of harvest index, (kg/ha)/(kg/ha)
USLE_C	Minimum C factor	Minimum value of USLE C factor for water erosion applicable to the land cover/plant. no unit

GSI	Maximum stomatal conductance	Maximum stomatal conductance at high solar radiation and low vapor pressure deficit. (m·s-1)
VPDFR	Vapor pressure deficit	Vapor pressure deficit (kPa) corresponding to the second point on the stomatal conductance curve. (The first point on the stomatal conductance curve is comprised of a vapor pressure deficit of 1 kPa and the fraction of maximum stomatal conductance equal to 1.00.). (kPa)
FRGMAX	Max Stomatal Conductance 2	Fraction of maximum stomatal conductance corresponding to the second point on the stomatal conductance curve. (kPa)
WAVP	BIO_E decline	Rate of decline in radiation use efficiency per unit increase in vapor pressure deficit. no unit
CO2HI	Elevated CO2 concentration	Elevated CO2 atmospheric concentration (μL CO2/L air) corresponding the 2nd point on the radiation use efficiency curve. (μL CO2/L air)
BIOEHI	CO2HI biomass energy ratio	Biomass-energy ratio corresponding to the 2nd point on the radiation use efficiency curve. no unit
RSDCO_PL	Daily Residue Decomposition	Plant residue decomposition coefficient. no unit
ALAI_MIN	Minimum Leaf Area Index	Minimum leaf area index for plant during dormant period. (m2 /m2)
BIO_LEAF	Biomass Fraction Leaf	Fraction of tree biomass accumulated each year that is converted to residue during dormancy. no unit
MAT_YRS	Tree Years Maturity	Number of years required for tree species to reach full development. (years)
BMX_TRE ES	Forest Maximum Biomass	Maximum biomass for a forest. (metric tons/ha)
EXT_COEF	Light Extinction Coefficient	Light extinction coefficient. no unit
BMDIEOFF	Biomass Dieoff Fraction	This variable pertains to trees only. (The value is never used for other types of plants.) The maximum biomass for a mature forest stand generally falls in the range of 30-50 metric tons/ha. no unit
RSR1C	Root Shoot Ratio Initial	Initial root to shoot ration at the beginning of the growing season. no unit
RSR2C	Root Shoot Ratio Final	Root to shoot ration at the end of the growing season. no unit

Table S4 – Description of the planting and harvesting dates for each crop.

	Planting		Harvesting	
Crop	Month	Day	Month	Day
APPL	3	1	8	31
BLUE	4	25	10	15
BROC	5	20	7	17
CABG	5	10	6	20
CANA	4	27	10	10
CHER	3	15	8	15
COLG	5	10	6	20
CRRT	4	15	5	2
CUCM	4	20	5	28
DRYB	5	15	5	22
GPEA	4	1	7	3
GRAP	3	16	8	31
HMEL	4	25	5	17
KALE	5	10	6	20
LETT	4	20	6	15
ONIO	4	10	9	20
PEAR	3	1	9	1
POTA	4	25	9	10
PUMP	5	10	9	25
RASP	3	15	8	20
SCRN	5	5	9	15
SGBT	4	20	8	30
SNPB	4	15	8	20
SPIN	4	15	6	10
SPOT	5	1	10	1
SQUA	5	10	9	25
STRW	4	15	8	15
TOMA	5	1	9	15
WWHT	10	1	7	15

References

Amon-Armah F, Yiridoe EK, Ahmad NHM, et al. (2013) Effect of nutrient management planning on crop yield, nitrate leaching and sediment loading in Thomas Brook watershed. Environ Manage 52:1177–1191. https://doi.org/10.1007/s00267-013-0148-z

Akhavan S, Abedi-Koupai J, Mousavi SF, et al. (2010) Application of SWAT model to investigate nitrate leaching in Hamadan-Bahar Watershed, Iran. Agric Ecosyst Environ 139:675–688. https://doi.org/10.1016/j.agee.2010.10.015

Babel MS, Gunathilake MB, Jha MK (2021) Evaluation of ecosystem-based adaptation measures for sediment yield in a tropical watershed in Thailand. Water (Switzerland) 13. https://doi.org/10.3390/w13192767

Bulut E, Aksoy A (2008) Impact of fertilizer usage on phosphorus loads to Lake Uluabat. Desalination 226:289–297. https://doi.org/10.1016/j.desal.2007.02.112

Cakir R, Sauvage S, Gerino M, et al. (2020) Assessment of ecological function indicators related to nitrate under multiple human stressors in a large watershed. Ecol Indic 111. https://doi.org/10.1016/j.ecolind.2019.106016

Curk M, Glavan M, Pintar M (2020) Analysis of nitrate pollution pathways on a vulnerable agricultural plain in Slovenia: Taking the local approach to balance ecosystem services of food and water. Water (Switzerland) 12:1–15. https://doi.org/10.3390/w12030707

Delavar M, Eini MR, Kuchak VS, et al. (2022) Model-based water accounting for integrated assessment of water resources systems at the basin scale. Science of the Total Environment 830. https://doi.org/10.1016/j.scitotenv.2022.154810

Donmez C, Sari O, Berberoglu S, et al. (2020) Improving the applicability of the SWAT model to simulate flow and nitrate dynamics in a flat data-scarce agricultural region in the mediterranean. Water (Switzerland) 12:1–24. https://doi.org/10.3390/w12123479

dos Santos FM, de Souza Pelinson N, de Oliveira RP, Di Lollo JA (2023) Using the SWAT model to identify erosion prone areas and to estimate soil loss and sediment transport in Mogi Guaçu River basin in Sao Paulo State, Brazil. Catena (Amst) 222: https://doi.org/10.1016/j.catena.2022.106872

Eini MR, Salmani H, Piniewski M (2023) Comparison of process-based and statistical approaches for simulation and projections of rainfed crop yields. Agric Water Manag 277. https://doi.org/10.1016/j.agwat.2022.108107

Emami F, Koch M (2018) Agricultural water productivity-based hydro-economic modeling for optimal crop pattern and water resources planning in the Zarrine River Basin, Iran, in the wake of climate change. Sustainability (Switzerland) 10. https://doi.org/10.3390/su10113953

Gholami F, Nemati A, Li Y, Zhang J (2022) Calculation of runoff computation cost and sensitivity analysis of topological attributes. Remote Sens Appl 26. https://doi.org/10.1016/j.rsase.2022.100714

Gholami F, Sedighifar Z, Ghaforpur P, et al. (2023) Spatial-temporal analysis of various land use classifications and their long-term alteration's impact on hydrological components: using remote sensing, SAGA-GIS, and ARCSWAT model. Environ Sci (Camb) 9:1161–1181. https://doi.org/10.1039/d2ew00138a

Goudarzi FM, Sarraf A, Ahmadi H (2019) The effects of climate change on crop yields using RCP scenarios with SWAT agro-hydrological model in Maharlu Basin (Fars Province - Iran). International Journal of Water 13:348. https://doi.org/10.1504/IJW.2019.106048

Gunacti MC, Kandemir FA, Najar M, et al. (2022) Attribution of changes in the water balance of a basin to land-use changes through combined modelling of basin hydrology and land-use dynamics. Journal of Water and Climate Change 13:4087–4104. https://doi.org/10.2166/wcc.2022.304

Halecki W, Kruk E, Ryczek M (2018) Loss of topsoil and soil erosion by water in agricultural areas: A multi-criteria approach for various land use scenarios in the Western Carpathians using a SWAT model. Land use policy 73:363–372. https://doi.org/10.1016/j.landusepol.2018.01.041

Han Y, Liu Z, Chen Y, et al. (2023) Assessing non-point source pollution in an apple-dominant basin and associated best fertilizer management based on SWAT modeling. International Soil and Water Conservation Research 11:353–364. https://doi.org/10.1016/j.iswcr.2022.10.002

Huang J, Ridoutt BG, Zhang H, et al. (2014) Water footprint of cereals and vegetables for the Beijing market: Comparison between local and imported supplies. J Ind Ecol 18:40–48. https://doi.org/10.1111/jiec.12037

Huo A, Huang Z, Cheng Y, van Liew MW (2020) Comparison of two different approaches for sensitivity analysis in Heihe River basin (China). Water Sci Technol Water Supply 20:319–327. https://doi.org/10.2166/ws.2019.159

Jalali J, Ahmadi A, Abbaspour K (2021) Runoff responses to human activities and climate change in an arid watershed of central Iran. Hydrological Sciences Journal 66:2280–2297. https://doi.org/10.1080/02626667.2021.1985724

Jeyrani F, Morid S, Srinivasan R (2021) Assessing basin blue–green available water components under different management and climate scenarios using SWAT. Agric Water Manag 256:. https://doi.org/10.1016/j.agwat.2021.107074

Kang X, Qi J, Li S, Meng FR (2022) A watershed-scale assessment of climate change impacts on crop yields in Atlantic Canada. Agric Water Manag 269. https://doi.org/10.1016/j.agwat.2022.107680

Liang K, Jiang Y, Qi J, et al. (2020) Characterizing the impacts of land use on nitrate load and water yield in an agricultural watershed in Atlantic Canada. Science of the Total Environment 729. https://doi.org/10.1016/j.scitotenv.2020.138793

Liang K, Qi J, Liu EY, et al. (2019) Estimated potential impacts of soil and water conservation terraces on potato yields under different climate conditions. J Soil Water Conserv 74:225–234. https://doi.org/10.2489/jswc.74.3.225

Mararakanye N, Le Roux JJ, Franke AC (2022) Long-term water quality assessments under changing land use in a large semi-arid catchment in South Africa. Science of the Total Environment 818. https://doi.org/10.1016/j.scitotenv.2021.151670

Marcinkowski P, Kardel I, Płaczkowska E, et al. (2023) High-resolution simulated water balance and streamflow data set for 1951–2020 for the territory of Poland. Geosci Data J 10:195–207. https://doi.org/10.1002/gdj3.152

Martínez-Casasnovas JA, Ramos MC, Benites G (2016) Soil and Water Assessment Tool soil loss simulation at the sub-basin scale in the Alt Penedès-Anoia vineyard region (NE Spain) in the 2000s. Land Degrad Dev 27:160–170. https://doi.org/10.1002/ldr.2240

Mehdi B, Ludwig R, Lehner B (2015) Evaluating the impacts of climate change and crop land use change on streamflow, nitrates and phosphorus: A modeling study in Bavaria. J Hydrol Reg Stud 4:60–90. https://doi.org/10.1016/j.ejrh.2015.04.009

Napoli M, Orlandini S (2015) Evaluating the Arc-SWAT2009 in predicting runoff, sediment, and nutrient yields from a vineyard and an olive orchard in Central Italy. Agric Water Manag 153:51–62. https://doi.org/10.1016/j.agwat.2015.02.006

Nkwasa A, Chawanda CJ, Jägermeyr J, Van Griensven A (2022) Improved representation of agricultural land use and crop management for large-scale hydrological impact simulation in Africa using SWAT+. Hydrol Earth Syst Sci 26:71–89. https://doi.org/10.5194/hess-26-71-2022

Pan S, Liu D, Wang Z, et al. (2017) Runoff responses to climate and land use/cover changes under future scenarios. Water (Switzerland) 9. https://doi.org/10.3390/w9070475

Paul M, Rajib A, Negahban-Azar M, et al. (2021) Improved agricultural water management in data-scarce semi-arid watersheds: Value of integrating remotely sensed leaf area index in hydrological modeling. Science of the Total Environment 791. https://doi.org/10.1016/j.scitotenv.2021.148177

Piniewski M, Marcinkowski P, O'Keeffe J, et al. (2020) Model-based reconstruction and projections of soil moisture anomalies and crop losses in Poland. Theor Appl Climatol 140:691–708. https://doi.org/10.1007/s00704-020-03106-6

Raij-Hoffman I, Miller K, Paul G, et al. (2022) Modeling water and nitrogen dynamics from processing tomatoes under different management scenarios in the San Joaquin Valley of California. J Hydrol Reg Stud 43:. https://doi.org/10.1016/j.ejrh.2022.101195

Samimi M, Mirchi A, Townsend N, et al. (2022) Climate change impacts on agricultural water availability in the middle Rio Grande basin. J Am Water Resour Assoc 58:164–184. https://doi.org/10.1111/1752-1688.12988

Udias A, Pastori M, Malago A, et al. (2018) Identifying efficient agricultural irrigation strategies in Crete. Science of the Total Environment 633:271–284. https://doi.org/10.1016/j.scitotenv.2018.03.152

Venishetty V, Parajuli PB (2022) Assessment of BMPs by estimating hydrologic and water quality outputs using SWAT in Yazoo River Watershed. Agriculture (Switzerland) 12 https://doi.org/10.3390/agriculture12040477

Wabela K, Hammani A, Abdelilah T, et al. (2022) Optimization of irrigation scheduling for improved irrigation water management in Bilate Watershed, Rift Valley, Ethiopia. Water (Switzerland) 14:. https://doi.org/10.3390/w14233960

Xue J, Huo Z, Kisekka I (2021) Assessing impacts of climate variability and changing cropping patterns on regional evapotranspiration, yield and water productivity in California's San Joaquin watershed. Agric Water Manag 250:. https://doi.org/10.1016/j.agwat.2021.106852

Yiridoe EK, Amon-Armah F, Hebb D, Jamieson R (2017) Eco-efficient choice of cropping system for reducing nitrate-N leaching in an agricultural watershed. Journal of Bioeconomics 19:201–221. https://doi.org/10.1007/s10818-016-9242-7

Zhang S, Liu Y, Wang T (2014) How land use change contributes to reducing soil erosion in the Jialing River Basin, China. Agric Water Manag 133:65–73. https://doi.org/10.1016/j.agwat.2013.10.016

Zhang Z, Wang Q, Guan Q, et al. (2023) Research on the optimal allocation of agricultural water and soil resources in the Heihe River Basin based on SWAT and intelligent optimization. Agric Water Manag 279:. https://doi.org/10.1016/j.agwat.2023.108177

Zorratipour M, Zarei H, Sharifi MR, Radmanesh F (2021) Hydrological simulation of Bakhtegan Basin in Iran using the SWAT model. Irrigation Sciences and Engineering 44:39–51. https://doi.org/10.22055/jise.2021.36821.1964

Zou M, Kang S, Niu J, Lu H (2018) A new technique to estimate regional irrigation water demand and driving factor effects using an improved SWAT model with LMDI factor decomposition in an arid basin. J Clean Prod 185:814–828. https://doi.org/10.1016/j.jclepro.2018.03.056
